# Efficacy and acceptability of different blood flow restriction training interventions during the rehabilitation of military personnel with lower limb musculoskeletal injuries: protocol for a two-phase randomised controlled trial

**DOI:** 10.1136/bmjopen-2024-096643

**Published:** 2025-05-26

**Authors:** Luke Gray, Russ J Coppack, Robert Barker-Davies, Robyn P Cassidy, Alexander N Bennett, Nick Caplan, Gavin Atkinson, Lauren Bradshaw, Janisha Chauhan, Kieran M Lunt, Luke Hughes, Peter Ladlow

**Affiliations:** 1 Department of Sport, Exercise and Rehabilitation, Northumbria University, Newcastle upon Tyne, Tyne and Wear, UK; 2 Academic Department of Military Rehabilitation, Defence Medical Rehabilitation Centre, Loughborough, UK; 3 Department for Health, University of Bath, Bath, UK; 4 School of Sport, Exercise, and Health Sciences, Loughborough University, Loughborough, UK; 5 Department of Bioengineering, Imperial College London, London, UK; 6 Defence Medical Rehabilitation Centre, Loughborough, UK

**Keywords:** Lower Extremity, SPORTS MEDICINE, REHABILITATION MEDICINE, Exercise

## Abstract

**Background:**

Musculoskeletal injury (MSKI) is the leading cause of medical downgrading and discharge within the UK military, with lower limb MSKI having the greatest incidence, negatively impacting operational readiness. Pain is a primary limiting factor to rehabilitation progress following MSKI. Heavy-load resistance training (RT; ie, loads >70% 1-repetition maximum) is traditionally used but may be contraindicated due to pain, potentially prolonging recovery and leading to failure of essential physical employment standards for UK military personnel. Low-load RT with blood flow restriction (BFR) can promote favourable morphological and physiological adaption, as well as elicit hypoalgesia in healthy and clinical populations (eg, post-operative), and has proven a viable option in military rehabilitation settings. The acceptability and tolerance of higher relative BFR pressures in persistent pain populations are unknown due to the complexity of presentation and the perception of discomfort experienced during BFR exercise. Greater relative pressures (ie, 80% limb occlusion pressure (LOP)) elicit a greater hypoalgesic response in pain-free individuals, but greater perceived discomfort which may not be tolerated in persistent pain populations. However, lower relative pressure (ie, 40% LOP) has elicited hypoalgesia in pain-free individuals, which therefore may be more clinically acceptable and tolerated in persistent pain populations. The primary aim of both randomised controlled trials (RCT) is to investigate the efficacy and acceptability of using high-frequency, low-load BFR-RT in UK military personnel with lower limb MSKI where persistent pain is the primary limiting factor for progression.

**Methodology:**

The presented protocol is a two-phase RCT based within a military rehabilitation setting. Phase One is a 1-week RCT to determine the most efficacious and acceptable BFR-RT protocol (7× BFR-RT sessions over 5 days at 40% or 80% LOP; n=28). Phase Two is a 3-week RCT comparing the most clinically acceptable BFR pressure, determined by Phase One (21× BFR-RT sessions over 15 days; n=26) to usual care within UK Defence Rehabilitation residential rehabilitation practices. Outcomes will be recorded at baseline, daily and following completion of the intervention. The primary outcome will be the brief pain inventory. Secondary outcomes include blood biomarkers for inflammation and pain (Phase Two only), injury-specific outcome measures, lower extremity function scale, objective measures of muscle strength and neuromuscular performance, and pressure pain threshold testing.

**Ethics and dissemination:**

The study is approved by the Ministry of Defence Research Ethics Committee (2318/MODREC/24) and Northumbria University. All study findings will be published in scientific peer-reviewed journals and presented at relevant scientific conferences.

**Trial registration number:**

Registered with Clinical Trials. The registration numbers are as follows: NCT06621914 (Phase One) and NCT06621953 (Phase Two).

**STRENGTHS AND LIMITATIONS OF THIS STUDY**

- This novel two-phase study is based within a military residential rehabilitation setting with real-world outcomes and implications.
- Our two-phase study compares high versus low pressure blood flow restriction with resistance training (BFR-RT) over a 1-week period, to assess the acceptability and efficacy, before implementing the most accepted and efficacious pressure during a 3-week rehabilitation intervention where BFR-RT and usual care are compared with usual care alone.
- Our study combines objective (eg, neuromuscular strength, pressure pain threshold and temporal summation, blood biomarkers) and subjective (eg, patient-reported outcome measures) outcome measures to assess the acceptability, effectiveness and tolerability of BFR-RT.
- Our study only includes short-term follow-ups due to the nature of the studies, and the setting it is embedded within (ie, quick deployment upon return to home-unit).

## Background

 The occupational requirements for serving military personnel include exposure to extreme, arduous and unpredictable environments, increasing the risk of musculoskeletal injury (MSKI).^1 2^ Within the UK military, MSKI is the leading cause of medical downgrading (ie, medically limited deployable or medically non-deployable) and discharge.^3^ Subsequently, this negatively impacts operational readiness and increases the demand on Defence Rehabilitation services, both at a substantial economic cost to the UK Ministry of Defence.^4 5^ Within the UK military, the greatest incidence of MSKI occurs at the lower limb,^1 6^ with lower limb MSKI medical discharge rates varying between 31 and 42% in 2022/2023.^7^ Pain is often considered a primary limiting factor to rehabilitation progress following MSKI, further delaying return-to-operational duty.^8^,^10^ Persistent pain is considered a frequent contributing factor to the rates of many MSKI-related medical downgrades/discharges across military populations.^3 11^

Persistent pain (defined as pain lasting >3 months)^12^ can occur innocuously (eg, fibromyalgia) or following injury (eg, post-surgical), resulting in pathology-specific pathophysiological changes that can cause life-changing, prolonged disability and loss of function, and poor long-term prognosis.^13 14^ Moreover, the development of persistent pain involves abnormal somatosensory processing in the central and/or peripheral nervous system.^15^ MSKI and associated pain and/or persistent pain are often associated with altered motor drive,^16^ kinesiophobia^17^ and symptomatic impairment,^18^ thus reducing rehabilitation potential, increasing recovery time and associated financial costs.^5^

UK Defence Rehabilitation currently offers 2-week and 3-week residential/inpatient rehabilitation courses at regional rehabilitation units, and the Defence Medical Rehabilitation Centre (DMRC), Stanford Hall, for more complex and/or chronic musculoskeletal conditions, including persistent pain populations. Residential rehabilitation courses are interdisciplinary in nature and have demonstrated efficacy for improving clinical outcomes in a wide array of MSKI.^19^,^21^ Rehabilitation outcomes are assessed against the physical employment standards with an increasing emphasis on the restoration of muscle strength and power alongside aerobic and anaerobic capacity, ensuring military personnel are physically prepared to tolerate and meet the physical demands of their occupational role.^22^

Within traditional rehabilitation settings, heavy-load resistance training (RT; ie, loads >70% 1-repetition maximum (RM)) is often mandated to develop muscular hypertrophy and strength to improve function following MSKI.^23 24^ However, persistent pain can create symptomatic impairment and thus contraindicate the use of heavy-load RT due to the risk of further injury and/or worsening of symptoms.^25 26^ An individual’s inability to produce high quantities of force or apply force rapidly due to MSKI and/or persistent pain prolongs the rehabilitation timeframe and leads to failure to meet the essential physical employment standards for UK military personnel.^5 22^ In addition, following an MSKI and/or persistent pain, a decrease in physical function and reduction in physical activity levels can occur, leading to progressive, disuse-induced atrophy of skeletal muscle.^27^ Consequently, a negative feedback loop occurs, whereby decreased functional status and physical activity levels promote further exacerbation of symptoms (eg, persistent pain, and movement-evoked pain) and skeletal muscle atrophy,^27 28^ further prolonging the duration of rehabilitation and risk of secondary health conditions.^29^

To combat the dearth of exercise rehabilitation interventions that attenuate pain, a proactive three-pronged approach is employed by Defence Medical Services (ie, detection, prevention and treatment).^30^ Defence Medical Services practitioners identified 11 research topics that should be prioritised, including novel and innovative treatments (eg, blood flow restriction (BFR) exercise), pain management in rehabilitation, rehabilitation outcomes and residential/inpatient treatment paradigm.^31^ Recent research suggests that BFR exercise can elicit hypoalgesia,^32^,^34^ as well as promote favourable physiological adaption in load-compromised populations.^35^,^37^ Ladlow *et al*^21^ reported comparable changes in muscular strength and hypertrophy following two times per day low-load RT with BFR (BFR-RT) when compared with traditional heavy-load RT, as well as a significant reduction in pain (BFR group only) in UK military personnel with lower limb MSKI, highlighting the efficacy of implementing BFR-RT within a military inpatient setting. Additionally, Mason *et al*^38^ investigated intensity-matched and volume-matched BFR versus a non-BFR group in active military personnel who had knee surgery (meniscal repair or chondral restoration) and reported a reduction in within-session anterior knee pain following BFR-RT, and comparable changes in patient-reported function and strength measures.

Hughes *et al*^33^ reported significant reductions in knee pain during and 24 hours following BFR-RT, but not traditional RT, throughout an 8-week intervention following anterior cruciate ligament reconstruction. Additionally, following 12 weeks of two times per week BFR-RT, a significant reduction in pain was noted in a knee osteoarthritis cohort.^35^ Furthermore, Giles *et al*^32^ reported a greater reduction in pain (p=0.02) during activities of daily living following 8 weeks of three times per week BFR-RT sessions compared with a traditional heavy-load RT control group in patients with anterior knee pain. Research specifically investigating BFR-induced hypoalgesia is still within its infancy and has been extrapolated from the work of exercise-induced hypoalgesia (EIH).^39 40^ However, to elicit an EIH response through traditional exercise modalities, high-intensity effort and/or longer durations of exercise are typically required (eg, >75% 1RM),^41^ which is often not feasible for load-compromised and/or persistent pain populations due to self-immobilisation and symptomatic impairments.^25 26^ The hypoalgesic response reported following BFR-RT occurs following low-load (20–30% 1RM) exercise.^32 33 35^ It has been proposed that a combination of factors accounts for the hypoalgesic response seen following BFR exercise, with the activation of opioid and endocannabinoid systems, conditioned pain modulation, high threshold motor unit recruitment, simulation of baroreceptors, and local mechanisms (eg, autoregulation of chemokine pathways, exercise metabolite accumulation, mast cell degranulation, and upregulation of anti-inflammatory cytokines), all being cited.^39 40 42^ Hughes *et al*,^43^ and Hughes and Patterson,^44^ reported a greater hypoalgesic response following low-intensity BFR exercise with a high pressure (ie, 80% of limb occlusion pressure (LOP)) compared with a lower pressure (ie, 40% LOP), and low-intensity exercise without BFR, suggesting a possible dose-response relationship between the pressure applied and magnitude of the hypoalgesic response. An acute increase in beta-endorphin, an endogenous neuropeptide, was reported in healthy participants following BFR-RT; however, at 24 hours, beta-endorphin levels were comparable to baseline, despite a local hypoalgesic response still being evident, suggesting that alternative mechanisms mediate BFR-induced hypoalgesia.^43 44^ This research supports the notion that local mechanisms play a substantial role in the hypoalgesic response of BFR exercise.^42^ Conversely, the EIH response is often inconsistent within persistent pain populations,^45^ with exercise sometimes eliciting hyperalgesia.^46^

A scarcity of literature is currently available concerning the impact of the applied pressure (ie, LOP) and BFR-induced hypoalgesia effect. The acceptability and tolerance of higher relative BFR pressures in persistent pain populations are also unknown due to the complexity of presentation (eg, hyperalgesia) and the perception of discomfort experienced during BFR exercise.^47 48^ Research currently suggests that greater pressures (ie, 80% LOP) elicit a greater hypoalgesic response in response to a noxious stimulus in pain-free individuals;^43 44^ however, if greater pressures are not tolerated, the opportunity to promote hypoalgesia could be lost, and it is currently unknown whether higher pressures elicit a greater acute hypoalgesic response in individuals with pain/persistent pain. Additionally, lower relative pressures (ie, 40% LOP) have elicited a statistically significant hypoalgesic response in pain-free individuals,^43 44^ which therefore may be more clinically acceptable and tolerated within persistent pain populations. Within UK Defence Rehabilitation residential rehabilitation courses, physiotherapists and exercise rehabilitation instructors only get a limited period per day with injured personnel; therefore, leveraging short-term pain reduction from BFR-RT may allow additional exercise rehabilitation activities to occur, akin to the work by Hughes *et al*,^33^ Korakakis *et al*^49^ and Ladlow *et al*.^21^ The clinical and mechanistic (biomechanical and neuromuscular) underpinnings of high-frequency BFR-RT in UK military personnel with persistent knee pain are currently being investigated at multiple regional rehabilitation units.^50^ However, this study does not directly measure mechanisms of pain modulation.

Any intervention that can accelerate the progression of complex MSKI rehabilitation and ameliorate pain, while exercising at a lower relative training intensity, is of interest to UK Defence Rehabilitation services.^50^ Research investigating the efficacy and acceptability of different BFR training interventions during the rehabilitation of military personnel with lower limb MSKI primarily limited by pain is warranted. To develop our understanding of BFR-induced hypoalgesia and develop best practice guidelines, a pragmatic research study, embedded within a ‘real-world’ clinical setting, is proposed.

## Study aims

The overall aim of both randomised controlled trials (RCT) is to investigate the efficacy and acceptability of using high-frequency, low-load BFR-RT in UK military personnel with lower limb MSKI where persistent pain is the primary limiting factor for progression.

### Phase One RCT

A 1-week pilot RCT will determine the most effective and acceptable BFR-RT protocol in UK military patients, with the primary aim of reducing pain. This will be achieved by comparing two different BFR-RT pressures (high-pressure (80% LOP) vs low-pressure (40% LOP)) within a military residential rehabilitation setting. It is hypothesised that high-pressure BFR-RT will elicit a greater hypoalgesic response, but be less tolerable (ie, less total work completed with higher perception of effect).

### Phase Two RCT

A 3-week RCT (resembling actual inpatient rehabilitation duration at DMRC) will compare the efficacy of the most clinically acceptable BFR pressure from Phase One to standard/conventional UK Defence Rehabilitation residential rehabilitation practice on physiological mechanisms underpinning changes in pain modulation and rehabilitation outcomes. It is hypothesised that the BFR-RT group will have greater reductions in pain, and increases in function, when compared with usual care alone.

## Methods and analysis: shared

### Study setting

Both phases of the study will be conducted at DMRC, Stanford Hall. The facility delivers 3-week residential rehabilitation courses to service personnel with complex/chronic injuries. The exercise rehabilitation components of each course are led by a physiotherapist and exercise rehabilitation instructor who receive specific training to ensure continuity, and best practice across UK Defence Rehabilitation. Details of course components are outlined in table 1. The experimental treatment group interventions will be delivered alongside a standardised rehabilitation programme (replacing the knee dominant exercises; leg press and knee extension).

**Table 1:** Components of residential rehabilitation at Defence Medical Rehabilitation Centre, Stanford Hall

*Course component*	*Intervention content*	*Intervention aim*	*Frequency and duration*
SEM/Rehabilitation Medicine Consultant Review	Clinical ReviewMedication ReviewPatient education for example, diagnostic description+/-support of imagingCo-ordination of investigation and referralLiaison with primary, secondary care and occupational health	Optimisation of health stateConsideration of co-morbiditiesConsideration of need for further investigations or imaging, for example, POCUS, MRI, X-ray etc.Consideration of need for further sub-specialist referral, for example, psychological support, neurophysiology, specialist pain teamRecommendations for occupational function and follow-up	Day one course reviewDischarge clinic on final day of courseInterim 1-2-1 or MDT course reviews as requiredTypical appointments 30 min
Individual Patient Assessment	MDT ClinicSubjective AssessmentObjective Assessment	Identify individual impairments and dysfunction to be addressed within the residential rehabilitation course	1× 1-hour session
Exercise-BasedTherapy (Group)	ERI led group-based training that involves cardiovascular exercise, functional movement patterns, hydrotherapy, minor team games, LL mobility, neuromuscular control, balance and proprioception, and LL strengthening	Improve muscle strength, and quality and timing of movement; increase joint range of motion; induce relaxation; promote normal walking gait; reduce pain levels	15× 45 min sessions
Exercise-BasedTherapy (Individual)	ERI/PT led individual, directly supervised, gym-based session compromising of elements of group-based session	As per group-based therapy	4× 1-hour sessions
Patient Education	Workshops and presentations covering anatomy and pathology of their lower limb injury, goal setting, nutrition, pain education science, planning and pacing, and principles of exercise	Improve ability to relax, and knowledge of self-help techniques and treatment options; improves patients understanding of injury, diagnosis and rehabilitation plan; promote behavioural change; reduction of pain; weight management	5× 30 min sessions
One-on-One Support	Individualised PT and/or OT sessions focusing on active/passive ROM exercises, advice on home exercise, cognitive behavioural therapy techniques, gait re-education training, manual therapy techniques, muscle activation and timing patterns, pain management, postural re-education, relaxation techniques, and self-help coping strategies	Control and reduce pain; improve muscle strength, and quality and timing of movement; increase joint ROM; induce relaxation; promote behavioural change and normal walking gait	5× 30 min sessions

ERI, exercise rehabilitation instructor; LL, lower limb; MDT, multidisciplinary team; OT, occupational therapy; POCUS, point-of-care ultrasound; PT, physiotherapy; ROM, range of movement; SEM, sport and exercise medicine.

### Ethics and dissemination

The study has been approved by the Army Scientific Advisory Committee and Ministry of Defence research ethics committee (2318/MODREC/24), Northumbria University, and is registered with clinicaltrials.gov (trial registration number: Phase One, NCT06621914; Phase Two, NCT06621953). The study sponsor is the Director of Research within UK Defence Medical Services. The study is jointly funded through the Defence Medical Services Research Steering Group, Delfi Medical Innovations Inc. and Northumbria University. The study protocol has been developed in accordance with the Standard Protocol Items for Randomised Trials (SPIRIT) statement^51^ (SPIRIT checklist found in online supplemental file 1) and the RCT will be delivered in accordance with Consolidated Standards of Reporting Trials (CONSORT) 2010 Statement: updated guidelines for reporting parallel group randomised trials.^52^ The results of these studies will be published by the investigators in relevant scientific peer-reviewed journals, regardless of study outcomes. Moreover, study results will be presented at relevant scientific conferences.

### Participants, recruitment and screening

All patients referred to DMRC, Stanford Hall multidisciplinary injury assessment clinic with MSKI of the lower limb with persistent pain as the primary limiting factor for progression will be screened against the eligibility criteria (table 2) by a sport and exercise medicine and/or rehabilitation medicine consultant within the lower limbs team who is not responsible for the direct treatment of the participant while on the residential rehabilitation course. Those who meet the eligibility criteria will be contacted via telephone by a member of the research team to discuss their possible inclusion in the study. Potential participants will be sent an information pack consisting of the patient information sheet. Participants who meet the eligibility criteria and provide written informed consent (returned to a member of the research team; online supplemental file 2) will be randomly assigned to one of the two study groups. Participants will then be invited to a pre-admission clinic to collect baseline outcome measures.

**Table 2:** Participant inclusion/exclusion criteria

*Inclusion*	*Exclusion*
Serving UK military personnel, Aged 18–55, Has unilateral lower limb injury whereby pain is the primary limiting factor hindering progression, as diagnosed by relevant consultant and team, Reduced occupational employability and function, Scheduled to attend Defence Medical Rehabilitation Centre, Stanford Hall, for 3-week residential rehabilitation course	Musculoskeletal-specific exclusion criteria Any medical contraindication related to blood flow restriction exercise* Non-musculoskeletal or serious pathological condition (ie, inflammatory arthropathy, infection or tumour) Spinal or referred pain from non-local pain source Any pre-diagnosed physical impairment or comorbidities (including cardiovascular disease) precluding the safe participation in the rehabilitation programme and/or assessment procedures Cortico-steroid or analgesic injection intervention to the affected area within the previous 7 days Currently pregnant or have not yet completed a return to work assessment following the birth of your child *Medical-related exclusion criteria History of cardiovascular disease (hypertension, peripheral vascular disease, thrombosis/embolism, ischaemic heart disease, myocardial infarction) History of the following musculoskeletal disorders: rheumatoid arthritis, avascular necrosis or osteonecrosis, severe osteoarthritis History of the following neurological disorders: Alzheimer’s disease, amyotrophic lateral sclerosis, peripheral neuropathy, Parkinson’s disease, severe traumatic brain injury Varicose veins in the lower limb Acute viral or bacterial upper or lower respiratory infection at screening Known or suspected lower limb chronic exertional compartment syndrome Surgical insertion of metal components at the position of cuff inflation History of any of the following conditions or disorders not previously listed: diabetes, active cancer History of elevated risk of unexplained fainting or dizzy spells during physical activity and/or exercise that causes loss of balance Increased risk of haemorrhagic stroke, exercise-induced rhabdomyolysis

### Randomisation and blinding procedure

For both phases, a permuted block randomisation method with a 1:1 ratio will be used using random block sizes. Randomisation will be stratified by age (18–36, 37–55 years old) and sex, to prevent an imbalance between groups.^53^ A plain language statement will inform participants that they have an equal chance of receiving low-load RT with either high-pressure or low-pressure BFR (Phase One), or low-load RT with BFR or standard rehabilitation (Phase Two). A sealed envelope will be opened to reveal group allocation by an independent administrator not involved in the recruitment, treatment or assessment of study outcomes. Given the nature of BFR, it is not feasible to blind participants to their treatment allocation. The clinical staff who deliver the study interventions and collect outcome data for the RCT must also be, by necessity, unblinded.

### Sample size calculation

For both phases, the sample size calculation was based on the effect size for a significant and clinically meaningful reduction in patient-reported pain (ie, the primary outcome measure, the Brief Pain Inventory (BPI))^54^ and calculated using G Power V.3.1.9.6.

Phase One: using a repeated measures analysis of variance (ANOVA; between factors) sample size calculation with an effect size of d=0.64,^54^ power=0.95, alpha=0.05, considering two treatment arms and three measurements timepoints (ie, pre-intervention and post-intervention, and follow-up), the required sample size is 24 patients. However, to account for a 10% drop out (as guided by previous ADMR publications^50 55 56^), a minimum of 28 patients will be recruited (n=14 per study arm).

Phase Two: using a repeated measures ANOVA (between factors) sample size calculation with an effect size of d=0.64,^54^ power=0.95, alpha=0.05, two treatment arms, and four measurements timepoints (ie, pre-intervention, end-week 1 intervention, end-week 2 intervention and post-intervention), the required sample size is 22 patients. However, to account for a 10% drop-out, 26 patients will be recruited (n=13 per study arm).

### Statistical methods and analysis

Descriptive data will be reported as the mean and SD for continuous variables and frequency statistics for non-continuous variables. Prior to statistical analysis, normality tests will establish data distribution; if data are non-normally distributed, transformation and the use of non-parametric statistical analysis tests will be employed. All tests will be two-sided, and alpha significant will be set a priori p<0.05. A between-subjects and within-subjects ANOVA will be used to assess the effect of the intervention on all outcome variables. Any statistically significant two-way interactions will be followed up using post hoc analysis with Bonferroni corrections to account for multiple comparisons. The magnitude of any differences will be presented using 95% CI and Cohen’s D for effect size.

Supporting analysis of the primary outcome will include a per-protocol analysis including patients with compliance >80% to intervention. In addition to the primary adjusted analysis, the unadjusted mean differences between groups will be reported using t-test, reporting 95% CI. Study participant flow will be recorded and reported as per CONSORT guidelines. Analysis will be conducted on a pairwise case basis. Therefore, all missing data will be reported, and patterns investigated. Sensitivity analysis will be conducted using multiple imputation techniques to assess the effect of missing data on primary outcomes. Post-intervention adverse events between groups will be analysed using Fisher’s exact test. All analysis will be conducted using SPSS.

### Data management

All data will be completed on hard copy by participants and collected by the research team. Participants will be identified through a unique identification key. Data access will be restricted to pre-identified researchers to ensure confidentiality. Only the research team involved in data analysis will have data access rights. All data collected will be stored securely at the study site. All data used in this project are crown copyright protected. On completion of the study, raw and processed data underpinning publications will be archived and stored securely on the electronic data archiving system at the Academic Department of Military Rehabilitation within the Ministry of Defence. Data will be retained for 10 years. This trial is embedded within an existing clinical care pathway, and as it is not testing new pharmaceutical products or drugs; therefore, a formal data monitoring committee was not required. However, a study steering group (LG, LH and PL) will meet periodically to discuss matters arising related to adherence and data management.

### Adverse events

All clinical and research staff will be briefed, detailing the procedures for identifying and reporting adverse events. Information on any unexpected adverse events deemed to be related to study participation will be collected and reported to the chief investigator within 24 hours of its occurrence. A standardised proforma will be completed by the study site clinician which will detail the time and date of the incident, severity of the event, the relationship to the study and the action taken and overall outcome. All serious adverse events will be recorded and discussed directly with the MODREC. Reporting of safety incidents will be duplicated using existing clinical health and safety reporting procedures and in accordance with the principles of good clinical practice. It is not anticipated that there will be any risk to study participants.

## Methods and analysis: Phase One

### Study design

**Figure 1:**
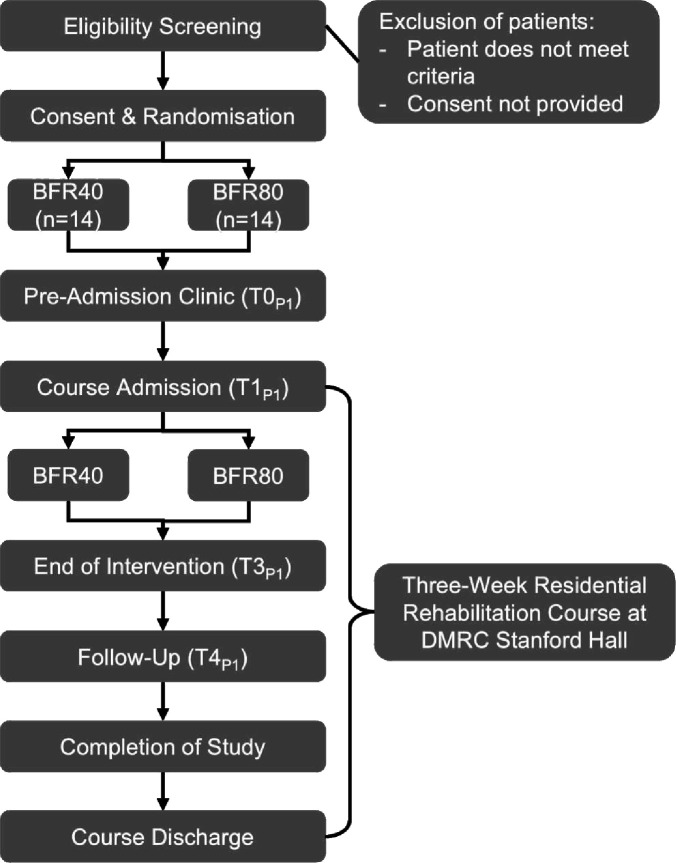
Study design flow diagram: Phase One. Phase One timepoints—T0_P1_, baseline (day −3); T1_P1_, admission day (day 1); T2_P1_, daily (days 1–5); T3_P1_, end of intervention (day 5); T4_P1_, follow-up (day 6). BFR40, blood flow restriction with resistance training at 40% limb occlusion pressure; BFR80, blood flow restriction with resistance training at 80% limb occlusion pressure; DMRC, Defence Medical Rehabilitation Centre.

Phase One is a 1-week, single-centre, pilot RCT embedded within DMRC, Stanford Hall’s residential rehabilitation courses, running from October 2024 to August 2025. Injured serving military personnel will be randomly assigned to one of the following groups: (1) low-load resistance training with high-pressure BFR (BFR80) or (2) low-load resistance training with low-pressure BFR (BFR40). Both treatment arms will be delivered during week 1 of the participant’s 3-week residential rehabilitation course. The study design is outlined in figure 1.

### Experimental intervention

During the first week of the residential rehabilitation course, participants in both groups will replace standardised knee dominant exercise with low-load BFR-RT at either low (ie, 40% LOP) or high (ie, 80% LOP) pressure, with previous literature reporting significant hypoalgesia following BFR exercise at both high and low pressures.^43 44^ Both experimental groups will complete seven BFR-RT sessions over the first week of the residential rehabilitation course (Monday–Friday). Using the Personalised Tourniquet System for BFR (Delfi Medical Innovations Inc, Vancouver BC, Canada), with an Easi-Fit BFR Cuff (single-bladder, contoured) fully encircling the limb and matching limb protection sleeve (24″×4.5″ or 34″×4.5″, dependent on limb size), each session will consist of a unilateral leg press exercise (Technogym, Bracknell, UK), followed by unilateral knee extension exercise with ankle weights (Komodo Sports, Huntingdon, UK), on the affected limb. The Personalised Tourniquet System for BFR automatically measures the individual’s LOP while at rest in supine (20–350 mm Hg±6 mm Hg of a set-point, 10 s average under non-transient conditions), which literature reports as a valid and reliable measure;^57^,^59^ thereafter, either 80% LOP (BFR80) or 40% LOP (BFR40) will be used based on the participants’ assigned group. Participants will complete 1 set of 30 repetitions, followed by 3 sets of 15 repetitions at 20% 1RM, with 30 s rest between sets (continuous inflation during exercise) and a 3 min reperfusion period between exercises. Both exercises will be performed using a 1:0:1 tempo (1 s concentric phase, no pause and 1 s eccentric phase). On the Wednesday and Thursday of the intervention week, BFR-RT will be performed twice daily, with sessions separated by >4 hours.

### Outcome measures

**Figure 2:**
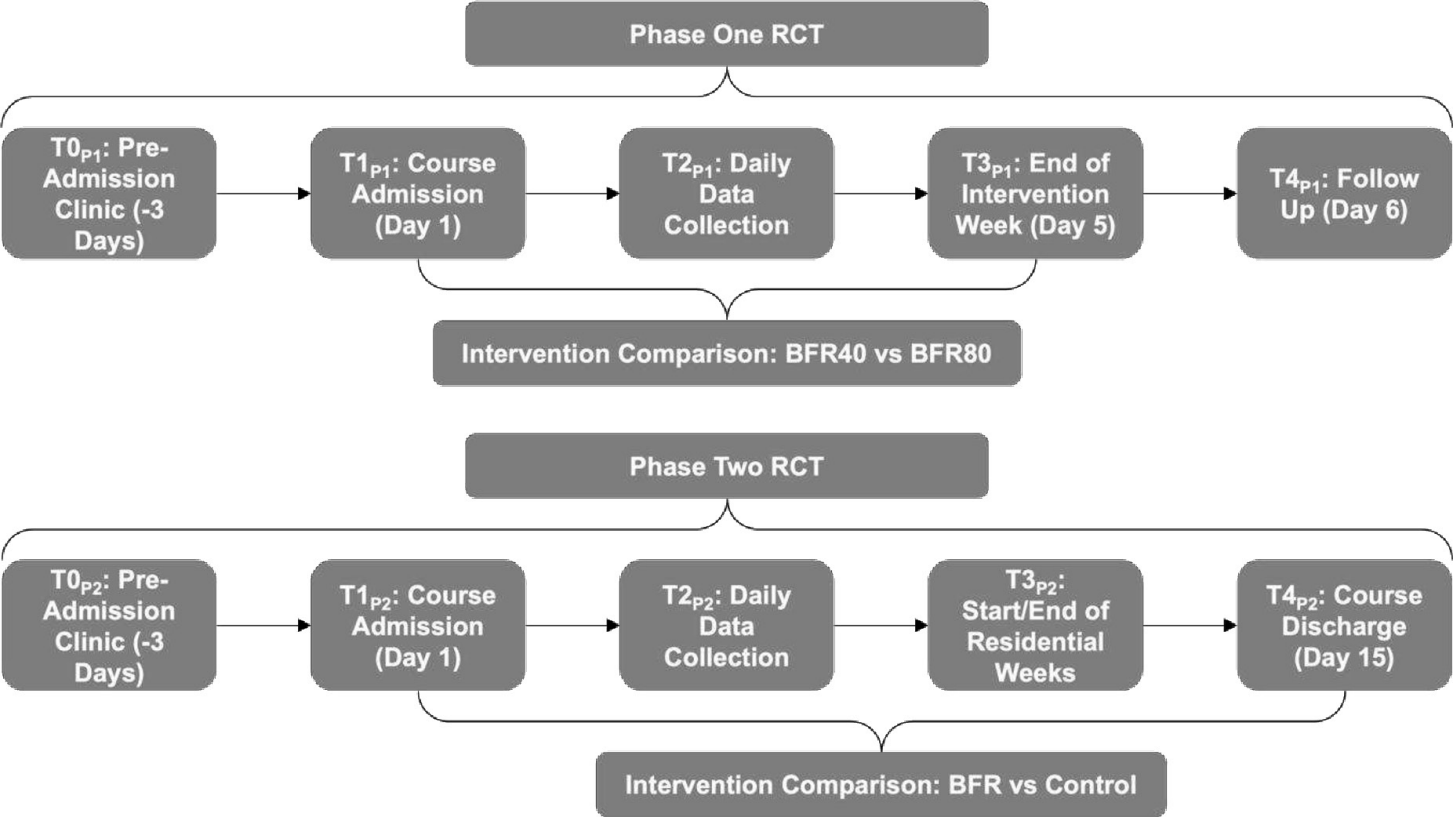
Timepoints of data collection for Phase One RCT (top) and Phase Two RCT (bottom). Phase One timepoints—T0_P1_, baseline (day −3); T1_P1_, admission day (day 1); T2_P1_, daily (days 1–5); T3_P1_, end of intervention (day 5); T4_P1_, follow-up (day 6). Phase Two timepoints—T0_P2_, baseline (day −3); T1_P2_, admission day (day 1); T2_P2_, daily (days 1–5, 6–10, 11–15); T3_P2_, start and end of residential rehabilitation weeks (days 1 and 5, 6 and 10, and 11 and 15); T4_P2_, end of intervention (day 15). BFR, blood flow restriction; BFR40, blood flow restriction with resistance training at 40% limb occlusion pressure; BFR80, blood flow restriction with resistance training at 80% limb occlusion pressure; RCT, randomised controlled trials.

A full list of outcome measures and their respective data collection timepoints are provided in online supplemental file 3, with an overview of each patient-reported outcome measure (PROM) provided in online supplemental file 4. A description of each timepoint (T0_P1_ to T4_P1_) is provided in figure 2.

#### Baseline characteristics

During Phase One, baseline data collection (T0_P1_) will include a participant demographics questionnaire, Health Anxiety Depression Scale,^60^ Musculoskeletal Health Questionnaire^61^ and medication history. Personal and demographic characteristics including age, body height, body mass, body mass index, duration of symptoms, previous injuries, previous treatment, military occupation, duration of military service, smoking and drinking habits will also be collected at T0_P1_.

#### Patient-reported outcome measures

The primary outcome measure of intervention efficacy will be the BPI;^62^ additionally, all participants will be asked to complete the Lower Extremity Function Scale (LEFS).^63^ A detailed description of all PROMs can be found in online supplemental file 4. All other PROMs listed will only be completed based on injury-specific site location. For example, an individual with a foot/ankle injury will only be asked to complete foot/ankle questionnaires, not knee or hip-specific questionnaires.

The BPI is a patient-administered, multidimensional, pain assessment tool commonly used within musculoskeletal clinical practice.^62^ Higher scores on the nine-item short form indicate greater interference with function, or greater pain intensity.^62^ Literature suggests the BPI has excellent test-retest reliability (intra-class correlation coefficient=0.90–0.96) and excellent internal consistency (Cronbach’s α=0.86–0.96) in populations with persistent neuropathic, nociceptive and nociplastic pain.^64^,^66^ The minimally clinically important difference for the BPI is a 2-point reduction for average pain, pain interference and pain severity.^67^,^69^

To assess the feasibility and tolerability of the BFR-RT interventions, a participant monitoring booklet will be used throughout the intervention period (T2_P1_). The booklet consists of multidimensional daily morning well-being questions, training load monitoring (sets × reps × load completed), sessional ratings of perceived exertion, and Numerical Pain Rating Scale (NPRS) for injury-specific pain and localised muscle soreness (NPRS; pre-intervention, immediately post-intervention and 1-hour post-intervention for each BFR-RT session).

#### Pressure pain threshold testing

Pressure pain threshold (PPT) testing is reported as the minimum pressure applied to cause pain and used to quantify pain sensitivity in persistent pain populations.^70^ It is suggested that PPT could be used as an indicator of persistent pain, as a reduction is consistent with hyperexcitability in central nervous system processing.^71^ The inter-rater and intra-rater reliability of PPT has previously been established as good to excellent, despite significant variation between measurement procedures being reported within the literature.^72^,^75^ For the collection of data within this pilot RCT, a member of research staff skilled in using a handheld pressure algometer (1 cm^2^ probe, Wagner Instruments, Greenwich, USA) will apply pressure at a rate of 1 kgf/s until the first point of perceived pain, whereby the participant will say ‘pain’ to indicate the perceived pain. The selected locations of measurement are based on existing literature:^43 44^

1. Bilateral quadriceps—test location measured 20 cm proximal to base of patella.
2. Dominant biceps brachii—test location measured 10 cm proximal to the cubital fossa.
3. Medial gastrocnemius of the injured/dominant limb—test location measured at 60% length of gastrocnemius muscle, measuring from calcaneus to popliteal fossa.
4. Nondominant upper trapezius—test location measured at 10 cm from the acromion in direct line with the neck.
5. Tibialis anterior of injured/dominant limb—test location measured at one-quarter of the distance between the superior edge of the fibular head and the most lateral part of the lateral malleolus was marked using a delible pen; then, half the distance from the palpated anterior-lateral edge of the tibia horizontal to the first mark is marked with a delible pen as testing location.

Pressure pain threshold testing will be completed at T0_P1_, T1_P1_, T2_P1_, T3_P1_ and T4_P1_. At each location, two measures will be collected, separated by 20 s, with the mean score for analysis (PPT quantified as the kilogram force applied at the point of ‘pain’). Thereafter, PPT testing will be completed pre-intervention, immediately post-intervention and 60 min post-intervention to assess the immediate and lasting effects of the intervention. Additionally, the pre-intervention PPT metrics on the following day will act as a 24-hour mechanistic measure of BFR-induced hypoalgesia following BFR intervention. As a result of assessing six separate points, we will be able to determine if the BFR interventions produce a systemic as well as a local hypoalgesic effect.

#### Physical and functional capacity assessment

##### 5RM leg press and knee extension

Multiple repetition strength assessments are associated with a lower risk of injury and symptoms of delayed muscle soreness as skeletal muscles, connective tissue and joints are exposed to lower loads than with maximal strength testing, such as 1RM;^76^ thus, 5RM testing is considered a more suitable assessment method within a rehabilitation setting for injured personnel. Therefore, unilateral muscle strength will be assessed using a dynamic 5RM test, defined as the maximal load (kg) that the participant can lift five times consecutively with the correct lifting technique. This will be performed on a leg press and knee extension machine to assess functional strength and is aligned with current clinical care practice. This test has demonstrated good test-retest reliability and can be used as a valid predictor of maximal strength.^77 78^ This outcome measure will be collected at T0_P1_ and T4_P1_, only, in Phase One.

##### Isometric mid-thigh pull

Isometric strength testing may provide a safer alternative for the quantification of force production relating to the elimination of painful joint movements under loaded conditions, thus offering clinical testing utility to those where pain is the primary limiting factor to performance.^79^,^81^ The isometric mid-thigh pull (IMTP) is currently implemented as a role fitness test within the British Army Physical Employment Standards, integrated into lower limb rehabilitation settings within UK Defence Rehabilitation,^79^ and has demonstrated good-to-excellent reliability in measuring maximal strength.^82^ The IMTP is a test that can assess multiple derivatives of maximal lower limb muscle force production capabilities, including peak force, rate of force development and limb symmetry, and will be delivered using a previously established, standardised testing procedure^80^ on a pair of portable force plates (Hawkin Dynamics, Portland, Maine, USA) located on the base plate of a mid-thigh pull rig (Absolute Performance, Cardiff, UK). Force-time data will be sampled at 1000 Hz and will be visually assessed against a previously established criteria, with invalid trials repeated.^80^ Isometric mid-thigh pull assessment will be completed at T1_P1_ and T4_P1_, in Phase One.

## Methods and analysis: Phase Two

### Study design

**Figure 3:**
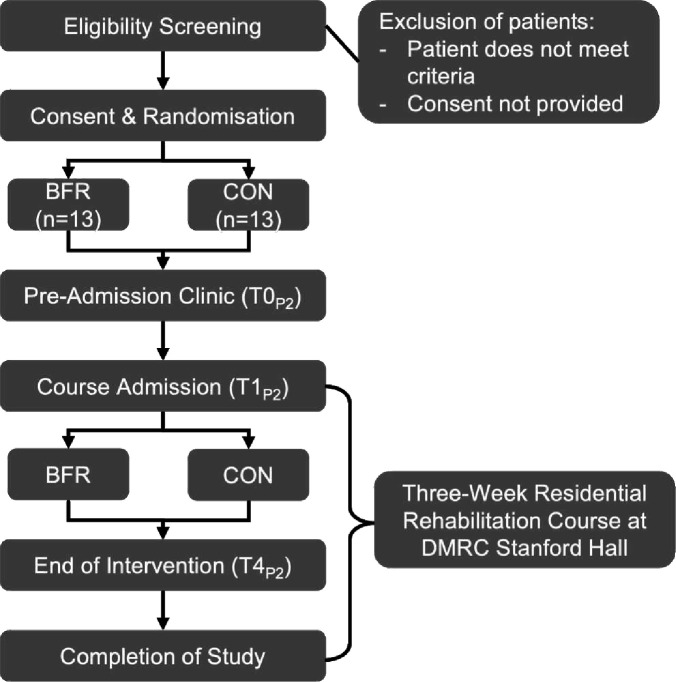
Study design flow diagram: Phase Two. Phase Two timepoints—T0_P2_, baseline (day −3); T1_P2_, admission day (day 1); T2_P2_, daily (days 1–5, 6–10, 11–15); T3_P2_, start and end of residential rehabilitation weeks (days 1 and 5, 6 and 10, and 11 and 15); T4_P2_, end of intervention (day 15). BFR, blood flow restriction with resistance training; CON, standard residential rehabilitation/usual care group; DMRC, Defence Medical Rehabilitation Centre.

Phase Two is a 3-week, single-centre RCT embedded within DMRC Stanford Hall’s residential rehabilitation courses running from August 2025 to May 2026. Injured serving military personnel will be randomly assigned to one of the following groups: (1) low-load resistance training with either high-pressure or low-pressure BFR as determined by the outcome of Phase One or (2) standard residential rehabilitation (CON). The experimental treatment arm will be delivered alongside the 3-week residential rehabilitation course. The study design is outlined in figure 3.

### Experimental intervention

Throughout the residential rehabilitation course, participants in the BFR group will have their standardised knee dominant exercises replaced with low-load BFR-RT at either high or low pressure. The decision as to which BFR pressure (ie, high or low pressure) will be implemented within Phase Two will be determined by a multistage process: (1) the pressure which elicits the greatest reduction in pain as per the primary outcome measure (BPI); (2) if no statistically significant difference is reported for pain, the pressure which promotes the greatest improvement in function as per the secondary outcome measure (LEFS) will be used; or (3) if no difference between groups for pain (BPI) or function (LEFS), the low-pressure group will be used due to greater tolerability and reduced perception of effort. The BFR group will complete 21× BFR-RT sessions over the 3-week residential rehabilitation course. Phase Two uses the same BFR protocol as in Phase One with only the number of sessions (21× vs 7× sessions) and study duration (3 weeks vs 1 week) changing. The control group will receive standard residential rehabilitation only, as described in table 1.

### Outcome measures

#### Baseline characteristics

The same baseline characteristics as Phase One will be collected in Phase Two, at T0_P2_.

#### Patient-reported outcome measures

The PROMs used in Phase One (BPI, LEFS and injury-specific) will also be used within Phase Two, collected at T0_P2_, T3_P2_ and T4_P2_. Additionally, secondary outcome measures will be collected, including the Fatigue Assessment Scale, McGill Pain Questionnaire – Short Form, Pain Catastrophising Scale and Tampa Scale of Kinesiophobia. These secondary outcome measures will be collected at T0_P2_ and T4_P2_ only. The assessment of tolerability and daily response will again be monitored using the participant monitoring booklet (T2_P2_). The data collection schedule for all outcome measures can be found in online supplemental file 3.

#### Mechanistic pain measures

##### Pressure pain threshold

Following the same protocol as per Phase One, PPTs will be collected at T0_P2_–T4_P2_.

##### Temporal summation

Temporal summation is a form of quantitative sensory testing that uses mechanical pressure to gain insight into which pain mechanisms are involved for an individual patient.^83^ When used at local and remote sites of injured tissue, the assessment of temporal summation tests the function of A and C fibres, and their associated central pathways, and identifies the presence of local (peripheral sensitisation) and/or centrally driven symptoms (eg, secondary hyperalgesia).^83^,^85^ Central sensitisation, defined as increased nociceptive responsiveness following normal or subthreshold afferent input, may occur in different areas of the nervous system (eg, dorsal horn neurons),^86^ and current evidence suggests temporal summation is indicative of central sensitisation.^87^ Temporal summation assessment resembles the ‘wind-up’ effect, or second pain stimulus,^86 88^ often seen in persistent pain populations, by stimulating the unmyelinated C fibres.^89 90^ A measure of temporal summation will be taken at T0_P2_ and T4_P2_. To assess temporal summation, at the location closest to the individuals’ injured site from the previously described PPT test locations, 10 consecutive ramping pulses building to the mean pressure of the respective PPT score will be applied over 5 s with a 1 s hold, before immediate release, with 1 s rest interval before the next pulse. At the peak of each pulse, the participant will be asked to rate their pain on the NPRS scale. The first NPRS value will act as an anchor/baseline. Thereafter, each NPRS will be normalised via subtraction of the initial NPRS value. To reflect changes in temporal summation across the series, the NPRS epochs (mean NPRS rating of peaks; I 2–4, II 5–7 and III 8–10) will be calculated for the statistical analysis.^91^

##### Blood biomarkers

To date, there is limited literature investigating the effect of high-frequency BFR-RT on markers of inflammation and hypoalgesia.^92^ During Phase Two, 19× 10 mL venous blood samples from an antecubital vein will be collected at various timepoints to assess the acute and chronic effects of BFR-RT on hypoalgesia and inflammation, respectively. Beta-endorphin is a neuropeptide produced in the central nervous system and pituitary gland in response to pain or exercise^93^ and shares a similar structure to morphine and the same binding sites within the brain cells and receptors, thereby working as a powerful analgesic by inhibiting the communication of pain signals.^93^ Additionally, beta-endorphin has previously been used as an outcome measure within the BFR literature, with elevated levels reported following BFR-RT and BFR with aerobic exercise immediately post-intervention.^43 44^ Venous blood samples for beta-endorphin will be collected at T0_P2_, T2_P2_ (BFR only: Tuesday pre-intervention, immediately post-intervention, and 1-hour post-intervention, and Wednesday pre-intervention), T3_P2_ and T4_P2_. Inflammatory cytokines interleukin-6 and tumour necrosis factor-⍺ are thought to be drivers for the development of pathological pain^94^,^96^ and have been shown to have elevated levels in persistent pain populations^97^,^100^ and will be collected at T0_P2_, T3_P2_ and T4_P2_. To minimise pre-analytical variability within these biomarkers, all samples will be obtained at the same time of day (08:00–12:00), after a ≥12-hour fast and abstinence from alcohol (≥24 hours) and caffeine (≥12 hours).^101^ All samples will be centrifuged at 1000 × g for 15 min, within 30 min of sample collection. Plasma will then be separated into 1.5 mL aliquots and stored in snap-seal microcentrifuge tubes (Sarstedt, Germany) at −80°C. Samples will be analysed at the biochemistry laboratory at Northumbria University using commercially available ELISA kits.

### Physical and functional capacity assessment

#### 5RM leg press and leg extension

Following the same protocol as per Phase One, 5RM assessment will take place at T0_P2_ and T4_P2_.

#### Isometric mid-thigh pull

Following the same protocol as per Phase One, IMTP assessment will take place at T0_P2_, T3_P2_ and T4_P2_.

## Discussion

The utility of BFR exercise is expanding across numerous clinical populations,^102^,^104^ with multisystem beneficial adaptions being increasingly reported (eg, cardiovascular, morphological and neural).^105^,^108^ Numerous studies have investigated BFR-RT utility over 6–8 weeks, with two to three sessions per week, mirroring a traditional rehabilitation approach, focusing on physiological adaption rather than pain.^37 109 110^ Additionally, Ladlow *et al*^21^ provided rationale for high-frequency usage within military rehabilitation settings; however, significantly less research has investigated high-frequency BFR-RT in inpatient setting when pain is the primary outcome measure. This two-phase research programme aims to expand upon the previous and currently ongoing UK Defence Rehabilitation RCT.^21 50^ To our knowledge, this two-phase RCT will be the first to assess the effect of high-frequency BFR-RT, and BFR-RT in general, on pain modulation in military personnel with lower limb MSKI. The clinical implications of these findings are that BFR-RT could be a rehabilitation intervention that can induce beneficial clinical adaptions and attenuate pain response, in the absence of high mechanical loads and pharmacological intervention, respectively. This study aims to optimise rehabilitation outcomes when hypoalgesia is the primary focus of treatment. If efficacy is established, BFR therapy for the management of MSKI associated pain could be applied across UK Defence Rehabilitation settings. The results of this study will provide insight and knowledge applicable to the wider clinical and scientific community, including those in civilian and professional sports settings.

### Methodological considerations and study limitations

Our participants are exclusively lower limb MSKI only; therefore, inference cannot be made to the efficacy of BFR-RT in upper limb injuries. Additionally, participants will comprise different diagnostic injury types and will be undergoing multimodal intervention, which may attenuate the treatment effect. The small sample sizes included within both phases limit the ability to make definitive statements regarding the effectiveness of BFR-RT, and results may be susceptible to type I or II errors. The lack of exercises-based control within Phase Two was deliberate, to assess whether BFR-RT offers additional value to usual care; however, we acknowledge that we therefore cannot say whether BFR-RT is more effective than traditional RT methods. Following completion of the residential rehabilitation course, personnel will return to their local units (nationwide) and could be deployed at short notice with limited/restricted duties. Therefore, a decision to exclude a follow-up assessment was made. The authors recognise this, which therefore means a conclusion to long-term benefit cannot be made.

## Supplementary material

10.1136/bmjopen-2024-096643 online supplemental file 1

10.1136/bmjopen-2024-096643 online supplemental file 2

10.1136/bmjopen-2024-096643 online supplemental file 3

10.1136/bmjopen-2024-096643 online supplemental file 4

## References

[1] Lovalekar M, Hauret K, Roy T (2021). Musculoskeletal injuries in military personnel-Descriptive epidemiology, risk factor identification, and prevention. J Sci Med Sport.

[2] Nindl BC, Castellani JW, Warr BJ (2013). Physiological Employment Standards III: physiological challenges and consequences encountered during international military deployments. Eur J Appl Physiol.

[3] Scott PJ, Peter M, Scott J (2021). Musculoskeletal injury outcomes: 2-year retrospective service evaluation of a UK defence primary care rehabilitation facility (PCRF). BMJ Mil Health.

[4] Hunter GR, McCarthy JP, Bamman MM (2004). Effects of resistance training on older adults. Sports Med.

[5] Andersen KA, Grimshaw PN, Kelso RM (2016). Musculoskeletal Lower Limb Injury Risk in Army Populations. *Sports Med Open*.

[6] Hayhurst D, Warner M, Stokes M (2024). Musculoskeletal injury in military specialists: a 2-year retrospective study. BMJ Mil Health.

[7] UK Ministry of Defence (2023). Annual medical discharges in the uk regular armed forces.

[8] El-Tallawy SN, Nalamasu R, Salem GI (2021). Management of Musculoskeletal Pain: An Update with Emphasis on Chronic Musculoskeletal Pain. Pain Ther.

[9] Rosenbloom BN, Khan S, McCartney C (2013). Systematic review of persistent pain and psychological outcomes following traumatic musculoskeletal injury. J Pain Res.

[10] Liew BXW, Del Vecchio A, Falla D (2018). The influence of musculoskeletal pain disorders on muscle synergies-A systematic review. PLoS ONE.

[11] Karasel S, Cebeci D, Sonmez I (2020). Chronic Pain and Pain Belief in Active Military Personnel: a Cross-sectional Study. *Med Arch*.

[12] Raffaeli W, Tenti M, Corraro A (2021). Chronic Pain: What Does It Mean? A Review on the Use of the Term Chronic Pain in Clinical Practice. J Pain Res.

[13] Chisala E, Bahadur S (2022). P176 Meeting the demands of employment: the impact and rehabilitation of chronic pain and fatigue in the UK armed forces. Rheumatology (Sunnyvale).

[14] Thapa P, Euasobhon P (2018). Chronic postsurgical pain: current evidence for prevention and management. *Korean J Pain*.

[15] Greene SA (2010). Chronic Pain: Pathophysiology and Treatment Implications. Top Companion Anim Med.

[16] Murphy MC, Rio EK, Whife C (2024). Maximising neuromuscular performance in people with pain and injury: moving beyond reps and sets to understand the challenges and embrace the complexity. BMJ Open Sport Exerc Med.

[17] Gulrandhe P, Warutkar V, Chitale N (2021). Fear avoidance model of kinesiophobia and rehabilitation. Journal of Medical Pharmaceutical and Allied Sciences.

[18] Farzad M, MacDermid JC, Mehta S (2021). Early post-immobilization pain at rest, movement evoked pain, and their ratio as potential predictors of pain and disability at six- and 12-months after distal radius fracture. *Arch Physiother*.

[19] Surtees JE, Heneghan NR (2020). General group exercise in low back pain management in a military population, a comparison with specific spine group exercise: a service evaluation. BMJ Mil Health.

[20] Coppack RJ, Bilzon JL, Wills AK (2016). Physical and functional outcomes following multidisciplinary residential rehabilitation for prearthritic hip pain among young active UK military personnel. *BMJ Open Sport Exerc Med*.

[21] Ladlow P, Coppack RJ, Dharm-Datta S Low-Load Resistance Training With Blood Flow Restriction Improves Clinical Outcomes in Musculoskeletal Rehabilitation: A Single-Blind Randomized Controlled Trial. Front Physiol.

[22] Orr RM, Dawes JJ, Lockie RG (2019). The Relationship Between Lower-Body Strength and Power, and Load Carriage Tasks: A Critical Review. Int J Exerc Sci.

[23] Kristensen J, Franklyn-Miller A (2012). Resistance training in musculoskeletal rehabilitation: a systematic review. *Br J Sports Med*.

[24] Reiman MP, Lorenz DS (2011). Integration of strength and conditioning principles into a rehabilitation program. Int J.

[25] Slysz J, Stultz J, Burr JF (2016). The efficacy of blood flow restricted exercise: A systematic review & meta-analysis. J Sci Med Sport.

[26] Hoyt BW, Pavey GJ, Pasquina PF (2015). Rehabilitation of Lower Extremity Trauma: a Review of Principles and Military Perspective on Future Directions. Curr Trauma Rep.

[27] Powers SK, Lynch GS, Murphy KT (2016). Disease-Induced Skeletal Muscle Atrophy and Fatigue. Med Sci Sports Exerc.

[28] Yin L, Li N, Jia W (2021). Skeletal muscle atrophy: From mechanisms to treatments. Pharmacol Res.

[29] Lorenz DS, Reiman MP, Walker JC (2010). Periodization: current review and suggested implementation for athletic rehabilitation. *Sports Health*.

[30] Woodgate P (2024). Realising the ambition of the Defence Medical Services research strategy. BMJ Mil Health.

[31] Coppack RJ, Ladlow P, Bennett AN (2022). Developing UK Defence Rehabilitation research priorities: a 2020 clinical practitioner engagement exercise. BMJ Mil Health.

[32] Giles L, Webster KE, McClelland J (2017). Quadriceps strengthening with and without blood flow restriction in the treatment of patellofemoral pain: a double-blind randomised trial. Br J Sports Med.

[33] Hughes L, Patterson SD, Haddad F (2019). Examination of the comfort and pain experienced with blood flow restriction training during post-surgery rehabilitation of anterior cruciate ligament reconstruction patients: A UK National Health Service trial. Phys Ther Sport.

[34] Korakakis V, Whiteley R, Epameinontidis K (2018). Blood Flow Restriction induces hypoalgesia in recreationally active adult male anterior knee pain patients allowing therapeutic exercise loading. Phys Ther Sport.

[35] Ferraz RB, Gualano B, Rodrigues R (2018). Benefits of Resistance Training with Blood Flow Restriction in Knee Osteoarthritis. Med Sci Sports Exerc.

[36] Vinolo-Gil MJ, Rodríguez-Huguet M, Martin-Vega FJ (2022). Effectiveness of Blood Flow Restriction in Neurological Disorders: A Systematic Review. Healthcare (Basel).

[37] Hughes L, Paton B, Rosenblatt B (2017). Blood flow restriction training in clinical musculoskeletal rehabilitation: a systematic review and meta-analysis. *Br J Sports Med*.

[38] Mason JS, Crowell MS, Brindle RA The Effect of Blood Flow Restriction Training on Muscle Atrophy Following Meniscal Repair or Chondral Restoration Surgery in Active Duty Military: A Randomized Controlled Trial. J Sport Rehabil.

[39] Hughes L, Patterson SD (2019). Low intensity blood flow restriction exercise: Rationale for a hypoalgesia effect. Med Hypotheses.

[40] Song JS, Spitz RW, Yamada Y (2021). Exercise-induced hypoalgesia and pain reduction following blood flow restriction: A brief review. Phys Ther Sport.

[41] Koltyn KF (2002). Exercise-Induced Hypoalgesia and Intensity of Exercise. Sports Med.

[42] Cervini GA, Rice M, Jasperse JL (2023). Potential Local Mechanisms for Exercise-Induced Hypoalgesia in Response to Blood Flow Restriction Training. *Cureus*.

[43] Hughes L, Grant I, Patterson SD (2021). Aerobic exercise with blood flow restriction causes local and systemic hypoalgesia and increases circulating opioid and endocannabinoid levels. J Appl Physiol.

[44] Hughes L, Patterson SD (2020). The effect of blood flow restriction exercise on exercise-induced hypoalgesia and endogenous opioid and endocannabinoid mechanisms of pain modulation. J Appl Physiol.

[45] Vaegter HB, Jones MD (2020). Exercise-induced hypoalgesia after acute and regular exercise: experimental and clinical manifestations and possible mechanisms in individuals with and without pain. Pain Rep.

[46] Rice D, Nijs J, Kosek E (2019). Exercise-Induced Hypoalgesia in Pain-Free and Chronic Pain Populations: State of the Art and Future Directions. J Pain.

[47] Mok E, Suga T, Sugimoto T (2020). Negative effects of blood flow restriction on perceptual responses to walking in healthy young adults: A pilot study. Heliyon.

[48] Wilson SH, Hellman KM, James D (2021). Mechanisms, Diagnosis, and Medical Management of Hyperalgesia: an Educational Review. Curr Anesthesiol Rep.

[49] Korakakis V, Whiteley R, Giakas G (2018). Low load resistance training with blood flow restriction decreases anterior knee pain more than resistance training alone. A pilot randomised controlled trial. Phys Ther Sport.

[50] Cassidy RP, Lunt KM, Coppack RJ (2023). ADAPTations to low load blood flow restriction exercise versus conventional heavier load resistance exercise in UK military personnel with persistent knee pain: protocol for the ADAPT study, a multi-centre randomized controlled trial. BMC Musculoskelet Disord.

[51] Chan A-W, Tetzlaff JM, Altman DG (2013). SPIRIT 2013 statement: defining standard protocol items for clinical trials. Ann Intern Med.

[52] Schulz KF, Altman DG, Moher D (2010). WITHDRAWN: CONSORT 2010 statement: Updated guidelines for reporting parallel group randomised trials. Int J Surg.

[53] Kernan WN, Viscoli CM, Makuch RW (1999). Stratified randomization for clinical trials. J Clin Epidemiol.

[54] Krebs EE, Bair MJ, Damush TM (2010). Comparative responsiveness of pain outcome measures among primary care patients with musculoskeletal pain. Med Care.

[55] Barker-Davies RM, Nicol A, McCurdie I (2017). Study protocol: a double blind randomised control trial of high volume image guided injections in Achilles and patellar tendinopathy in a young active population. BMC Musculoskelet Disord.

[56] Coppack RJ, Bilzon JL, Wills AK (2016). A comparison of multidisciplinary team residential rehabilitation with conventional outpatient care for the treatment of non-arthritic intra-articular hip pain in UK Military personnel - a protocol for a randomised controlled trial. BMC Musculoskelet Disord.

[57] Hughes L, Jeffries O, Waldron M (2018). Influence and reliability of lower-limb arterial occlusion pressure at different body positions. PeerJ.

[58] Hughes L, McEwen J (2021). Investigation of clinically acceptable agreement between two methods of automatic measurement of limb occlusion pressure: a randomised trial. BMC Biomed Eng.

[59] Masri BA, Day B, Younger ASE (2016). Technique for Measuring Limb Occlusion Pressure that Facilitates Personalized Tourniquet Systems: A Randomized Trial. J Med Biol Eng.

[60] Bjelland I, Dahl AA, Haug TT (2002). The validity of the Hospital Anxiety and Depression Scale. An updated literature review. *J Psychosom Res*.

[61] Hill JC, Kang S, Benedetto E (2016). Development and initial cohort validation of the Arthritis Research UK Musculoskeletal Health Questionnaire (MSK-HQ) for use across musculoskeletal care pathways. BMJ Open.

[62] Stanhope J (2016). Brief Pain Inventory review. OCCMED.

[63] Binkley JM, Stratford PW, Lott SA (1999). The Lower Extremity Functional Scale (LEFS): scale development, measurement properties, and clinical application. North American Orthopaedic Rehabilitation Research Network Phys Ther.

[64] Mendoza T, Mayne T, Rublee D (2006). Reliability and validity of a modified Brief Pain Inventory short form in patients with osteoarthritis. Eur J Pain.

[65] Kapstad H, Rokne B, Stavem K (2010). Psychometric properties of the Brief Pain Inventory among patients with osteoarthritis undergoing total hip replacement surgery. Health Qual Life Outcomes.

[66] Ferreira ACL, Pereira DS, da Silva SLA (2023). Validity and reliability of the short form brief pain inventory in older adults with nociceptive, neuropathic and nociplastic pain. Geriatr Nurs (Lond).

[67] Mathias SD, Crosby RD, Qian Y (2011). Estimating Minimally Important Differences for the Worst Pain Rating of the Brief Pain Inventory–Short Form. J Support Oncol.

[68] Mease PJ, Spaeth M, Clauw DJ (2011). Estimation of minimum clinically important difference for pain in fibromyalgia. *Arthritis Care & Research*.

[69] Song CY, Chen CH, Chen TW (2022). Assessment of Low Back Pain: Reliability and Minimal Detectable Change of the Brief Pain Inventory. Am J Occup Ther.

[70] Fischer AA (1986). Pressure Threshold Measurement for Diagnosis of Myofascial Pain and Evaluation of Treatment Results. Clin J Pain.

[71] Amiri M, Alavinia M, Singh M (2021). Pressure Pain Threshold in Patients with Chronic Pain: A Systematic Review and Meta-Analysis. Am J Phys Med Rehabil.

[72] Trouvin AP, Attal N, Perrot S (2022). Assessing central sensitization with quantitative sensory testing in inflammatory rheumatic diseases: A systematic review. Joint Bone Spine.

[73] Park G, Kim CW, Park SB (2011). Reliability and Usefulness of the Pressure Pain Threshold Measurement in Patients with Myofascial Pain. *Ann Rehabil Med*.

[74] Walton DM, Levesque L, Payne M (2014). Clinical Pressure Pain Threshold Testing in Neck Pain: Comparing Protocols, Responsiveness, and Association With Psychological Variables. Phys Ther.

[75] Bhattacharyya A, Hopkinson LD, Nolet PS (2023). The reliability of pressure pain threshold in individuals with low back or neck pain: a systematic review. Br J Pain.

[76] Gail S, Künzell S (2014). Reliability of a 5-Repetition Maximum Strength Test in Recreational Athletes. Dtsch Z Sportmed.

[77] Dohoney P, Chromiak JA, Lemire D (2002). Prediction of one repetition maximum (1-RM) strength from a 4-6 RM and a7-10 RM submaximal strength test in healthy young adult males. J Exerc Physiol.

[78] Reynolds JM, Gordon TJ, Robergs RA (2006). Prediction of one repetition maximum strength from multiple repetition maximum testing and anthropometry. J Strength Cond Res.

[79] Walters V, Coppack RJ, Cassidy RP (2022). Use of an isometric mid-thigh pull test during musculoskeletal rehabilitation: can the criterion values from the updated British Army physical employment standards be used to inform UK Defence Rehabilitation practice?. BMJ Mil Health.

[80] Comfort P, Dos’Santos T, Beckham GK (2019). Standardization and Methodological Considerations for the Isometric Midthigh Pull. Strength Cond J.

[81] De Witt JK, English KL, Crowell JB (2018). Isometric Midthigh Pull Reliability and Relationship to Deadlift One Repetition Maximum. J Strength Cond Res.

[82] Grgic J, Scapec B, Mikulic P (2022). Test-retest reliability of isometric mid-thigh pull maximum strength assessment: a systematic review. Biol Sport.

[83] Vardeh D, Mannion RJ, Woolf CJ (2016). Towards a mechanism-based approach to pain diagnosis. The journal of pain: official journal of the American Pain Society.

[84] Woolf CJ (2011). Central sensitization: Implications for the diagnosis and treatment of pain. *Pain*.

[85] Cruz-Almeida Y, Fillingim RB (2014). Can Quantitative Sensory Testing Move Us Closer to Mechanism-Based Pain Management?. *Pain Med*.

[86] Latremoliere A, Woolf CJ (2009). Central sensitization: a generator of pain hypersensitivity by central neural plasticity. J Pain.

[87] Castelo-Branco L, Cardenas-Rojas A, Rebello-Sanchez I Temporal Summation in Fibromyalgia Patients: Comparing Phasic and Tonic Paradigms. Front Pain Res.

[88] Staud R, Vierck CJ, Cannon RL (2001). Abnormal sensitization and temporal summation of second pain (wind-up) in patients with fibromyalgia syndrome. Pain.

[89] O’Brien AT, Deitos A, Triñanes Pego Y (2018). Defective Endogenous Pain Modulation in Fibromyalgia: A Meta-Analysis of Temporal Summation and Conditioned Pain Modulation Paradigms. J Pain.

[90] Nielsen J, Arendt-Nielsen L (1998). The importance of stimulus configuration for temporal summation of first and second pain to repeated heat stimuli. *Eur J Pain*.

[91] McPhee M, Graven-Nielsen T (2019). Alterations in Temporal Summation of Pain and Conditioned Pain Modulation Across an Episode of Experimental Exercise-Induced Low Back Pain. J Pain.

[92] de Queiros VS, Rolnick N, de Alcântara Varela PW (2022). Physiological adaptations and myocellular stress in short-term, high-frequency blood flow restriction training: A scoping review. PLoS One.

[93] Jain A, Mishra A, Shakkarpude J (2019). Beta endorphins: The natural opioids. ~ 323 ~. International Journal of Chemical Studies.

[94] Tanaka T, Narazaki M, Kishimoto T (2014). IL-6 in inflammation, immunity, and disease. Cold Spring Harb Perspect Biol.

[95] Zhou Y-Q, Liu Z, Liu Z-H (2016). Interleukin-6: an emerging regulator of pathological pain. J Neuroinflammation.

[96] Idriss HT, Naismith JH (2000). TNF alpha and the TNF receptor superfamily: structure-function relationship(s). Microsc Res Tech.

[97] Ohtori S, Miyagi M, Eguchi Y (2012). Efficacy of epidural administration of anti-interleukin-6 receptor antibody onto spinal nerve for treatment of sciatica. Eur Spine J.

[98] Nash D, Hughes MG, Butcher L (2023). IL-6 signaling in acute exercise and chronic training: Potential consequences for health and athletic performance. Scand J Med Sci Sports.

[99] Wang H, Glauben R, Gebhard K (2006). Orthopaedic proceedings.

[100] Gevers-Montoro C, Puente-Tobares M, Monréal A (2023). Urinary TNF-α as a potential biomarker for chronic primary low back pain. Front Integr Neurosci.

[101] Kackov S, Simundic AM, Gatti-Drnic A (2013). Are patients well informed about the fasting requirements for laboratory blood testing?. Biochem Med (Zagreb).

[102] Reina-Ruiz ÁJ, Martínez-Cal J, Molina-Torres G (2023). Effectiveness of Blood Flow Restriction on Functionality, Quality of Life and Pain in Patients with Neuromusculoskeletal Pathologies: A Systematic Review. Int J Environ Res Public Health.

[103] Angelopoulos P, Tsekoura M, Mylonas K (2023). The effectiveness of blood flow restriction training in cardiovascular disease patients: A scoping review. J Frailty Sarcopenia Falls.

[104] Jønsson AB, Krogh S, Laursen HS (2024). Safety and efficacy of blood flow restriction exercise in individuals with neurological disorders: A systematic review. Scandinavian Med Sci Sports.

[105] Centner C, Wiegel P, Gollhofer A (2019). Effects of Blood Flow Restriction Training on Muscular Strength and Hypertrophy in Older Individuals: A Systematic Review and Meta-Analysis. Sports Med.

[106] Centner C, Lauber B (2020). A Systematic Review and Meta-Analysis on Neural Adaptations Following Blood Flow Restriction Training: What We Know and What We Don’t Know. Front Physiol.

[107] Sardeli AV, Ferreira MLV, Santos LC (2022). Cardiovascular responses during and after aerobic and strength exercises with blood flow restriction in older adults. Sci Sports.

[108] Davids CJ, Raastad T, James LP (2021). Similar Morphological and Functional Training Adaptations Occur Between Continuous and Intermittent Blood Flow Restriction. J Strength Cond Res.

[109] Cuyul-Vásquez I, Leiva-Sepúlveda A, Catalán-Medalla O (2020). The addition of blood flow restriction to resistance exercise in individuals with knee pain: a systematic review and meta-analysis. Braz J Phys Ther.

[110] Li S, Shaharudin S, Abdul Kadir MR (2021). Effects of Blood Flow Restriction Training on Muscle Strength and Pain in Patients With Knee Injuries: A Meta-Analysis. Am J Phys Med Rehabil.

