# Supplementary material for: Efficacy and acceptability of different blood flow restriction training interventions during the rehabilitation of military personnel with lower limb musculoskeletal injuries: protocol for a two-phase randomised controlled trial
Source: BMJ Open. 2025 May 26;15(5):e096643. doi: 10.1136/bmjopen-2024-096643 (PMC12107567; doi:10.1136/bmjopen-2024-096643)
Supplement: online supplemental file 2 [file bmjopen-15-5-s002.docx]

| Phase One RCT | | | |
| --- | --- | --- | --- |
| Title of Study:  The LIMITLESS Pilot Study - The acute effects of blood flow restriction training for rehabilitation of military patients with lower limb musculoskeletal injuries primarily limited by pain: a pilot randomised controlled trial (Part 1).    **MODREC Reference:** 2318/MODREC/24 | | | |
|  | Please Initial or Tick Boxes | | |
| - - - 1. The nature, aims and risks of the research have been explained to me. I have read and understood the Participant Information Sheet and understand what is expected of me. All my questions have been answered fully to my satisfaction. |  |  |  |
| - - - 1. I understand that if I decide at any time during the research that I no longer wish to participate in this project, I can notify the researchers involved and be withdrawn from it immediately without having to give a reason. I also understand that I may be withdrawn from the study at any time by the research team. In neither case will this be held against me in subsequent dealings with the Ministry of Defence. |  |  |  |
| - - - 1. I understand that the screening process to decide if I am suitable to be selected as a participant may include completing a medical screening questionnaire and/or a physical examination by a Medical Officer and I consent to this*.* |  |  |  |
| - - - 1. I consent to the processing of my personal information for the purposes of this research study. I understand that such information will be treated as confidential and handled in accordance with the provisions of the Data Protection Act 2018 and UK GDPR. |  |  |  |
| - - - 1. This consent is specific to the particular study described in the Participant Information Sheet and shall not be taken to imply my consent to participate in any subsequent or future study from that detailed here. Upon reasonable request, grouped data (i.e., data that cannot individually identify you) may be shared to other research groups for the advancement of research and clinical practices, by the chief investigator.       2. In the result of medical findings of concern, I consent to the findings being shared with my GP or Medical Officer. |  |  |  |
| - - - 1. I understand that in the event of my sustaining injury, illness or death as a direct result of participating as a volunteer in this research, I or my dependants may enter a claim with the Ministry of Defence for compensation under the provisions of the No-Fault Compensation Scheme, details of which are attached. |  |  |  |
| - - - 1. **I agree to participate in this study.** |  |  |  |
| Participant’s Statement:  I **……………………………………………………**  agree that the research project named above has been explained to me to my satisfaction, and I agree to take part in the study. Signed : Date: | | | |
| Investigator’s Statement:  I **……………………………………………………**  confirm that I have carefully explained the nature, demands and any foreseeable risks of the proposed research to the Participant.  Signed : Date: | | | |
| **Contact Details of Chief Investigator:**  Name: Dr Peter Ladlow PhD  Address: Stanford Hall, DMRC, Stanford-on-Soar, Loughborough, LE12 5BL  Tel No: +44 (0) 1509251500 (ext: 3407)  E-mail: peter.ladlow100@mod.gov.uk | | | |
| **Contact Details of Independent Medical Officer or Volunteer Advocate:**  Name: Dr Shreshth Dharm-Datta MSc(SEM) MRCS MFSEM FEBPRM  Address: Stanford Hall, DMRC, Stanford-on-Soar, Loughborough, LE12 5BL  Tel No: +44 (0) 1509 251 500  E-mail: [shreshth.dharm-datta357@mod.gov.uk](mailto:shreshth.dharm-datta357@mod.gov.uk) | | | |
|  | | | |

| Phase Two RCT | | | |
| --- | --- | --- | --- |
| Title of Study:  The LIMITLESS Study - The effectiveness of blood flow restriction training for the rehabilitation of military patients with lower limb musculoskeletal injuries limited by pain: a Ministry of Defence randomised controlled trial (Part 2).    **MODREC Reference:** 2318/MODREC/24 | | | |
|  | Please Initial or Tick Boxes | | |
| - - - 1. The nature, aims and risks of the research have been explained to me. I have read and understood the Participant Information Sheet and understand what is expected of me. All my questions have been answered fully to my satisfaction. |  |  |  |
| - - - 1. I understand that if I decide at any time during the research that I no longer wish to participate in this project, I can notify the researchers involved and be withdrawn from it immediately without having to give a reason. I also understand that I may be withdrawn from the study at any time by the research team. In neither case will this be held against me in subsequent dealings with the Ministry of Defence. |  |  |  |
| - - - 1. I understand that the screening process to decide if I am suitable to be selected as a participant may include completing a medical screening questionnaire and/or a physical examination by a Medical Officer and I consent to this*.* |  |  |  |
| - - - 1. I consent to the processing of my personal information for the purposes of this research study. I understand that such information will be treated as confidential and handled in accordance with the provisions of the Data Protection Act 2018 and UK GDPR.       2. I give consent for research staff at DMRC to collect 20ml of blood from my arm. I understand that this sample will be stored at DMRC before being analysed at the University of Northumbria biochemistry laboratories, and anonymised data shared. After blood analysis is complete, I agree to my blood sample being destroyed. |  |  |  |
| - - - 1. This consent is specific to the particular study described in the Participant Information Sheet and shall not be taken to imply my consent to participate in any subsequent or future study from that detailed here. Upon reasonable request, grouped data (i.e., data that cannot individually identify you) may be shared to other research groups for the advancement of research and clinical practices, by the chief investigator.       2. In the result of medical findings of concern, I consent to the findings being shared with my GP or Medical Officer. |  |  |  |
| - - - 1. I understand that in the event of my sustaining injury, illness or death as a direct result of participating as a volunteer in this research, I or my dependants may enter a claim with the Ministry of Defence for compensation under the provisions of the No-Fault Compensation Scheme, details of which are attached. |  |  |  |
| - - - 1. **I agree to participate in this study.** |  |  |  |
| Participant’s Statement:  I **……………………………………………………**  agree that the research project named above has been explained to me to my satisfaction, and I agree to take part in the study. Signed : Date: | | | |
| Investigator’s Statement:  I **……………………………………………………**  confirm that I have carefully explained the nature, demands and any foreseeable risks of the proposed research to the Participant.  Signed : Date: | | | |
| **Contact Details of Chief Investigator:**  Name: Dr Peter Ladlow PhD  Address: Stanford Hall, DMRC, Stanford-on-Soar, Loughborough, LE12 5BL  Tel No: +44 (0) 1509251500 (ext: 3407)  E-mail: peter.ladlow100@mod.gov.uk | | | |
| **Contact Details of Independent Medical Officer or Volunteer Advocate:**  Name: Dr Shreshth Dharm-Datta MSc(SEM) MRCS MFSEM FEBPRM  Address: Stanford Hall, DMRC, Stanford-on-Soar, Loughborough, LE12 5BL  Tel No: +44 (0) 1509 251 500  E-mail: [shreshth.dharm-datta357@mod.gov.uk](mailto:shreshth.dharm-datta357@mod.gov.uk) | | | |
|  | | | |
