# Supplementary material for: Efficacy and acceptability of different blood flow restriction training interventions during the rehabilitation of military personnel with lower limb musculoskeletal injuries: protocol for a two-phase randomised controlled trial
Source: BMJ Open. 2025 May 26;15(5):e096643. doi: 10.1136/bmjopen-2024-096643 (PMC12107567; doi:10.1136/bmjopen-2024-096643)
Supplement: online supplemental file 3 [file bmjopen-15-5-s003.docx]

| **Supplementary File 3.** Data Collection Schedule | | | | | | | | | | |
| --- | --- | --- | --- | --- | --- | --- | --- | --- | --- | --- |
| *Domain/Outcome Measure* | *Phase One* | | | | | *Phase Two* | | | | |
|  | T0  _P1_ | T1  _P1_ | T2 _P1_ | T3 _P1_ | T4 _P1_ | T0 _P2_ | T1 _P2_ | T2 _P2_ | T3 _P2_ | T4 _P2_ |
| **Baseline Assessment** |  |  |  |  |  |  |  |  |  |  |
| - Demographics Questionnaire | X |  |  |  |  | X |  |  |  |  |
| - Health Anxiety Depression Scale (HADS) | X |  |  |  |  | X |  |  |  |  |
| - Musculoskeletal Health Questionnaire (MSK-HQ) | X |  |  |  |  | X |  |  |  |  |
| - Medication Record Sheet | X |  |  |  |  | X |  |  |  |  |
| **Patient Reported Outcomes** |  |  |  |  |  |  |  |  |  |  |
| *Function* |  |  |  |  |  |  |  |  |  |  |
| - Copenhagen Hip and Groin Outcome Score (HAGOS)* | X |  |  | X | X | X |  |  | X | X |
| - Foot and Ankle Disability Index (FADI)* | X |  |  | X | X | X |  |  | X | X |
| - Knee Injury and Osteoarthritis Outcome Score (KOOS)* | X |  |  | X | X | X |  |  | X | X |
| - Leeds Assessment of Neuropathic Symptoms and Signs (S-LANSS)* | X |  |  | X | X | X |  |  | X | X |
| - Lower Extremity Function Scale (LEFS) | X |  |  | X | X | X |  |  | X | X |
| - Non-Arthritic Hips Score (NAHS)* | X |  |  | X | X | X |  |  | X | X |
| - Victorian Institute Assessment – Achilles (VISA-A)* | X |  |  | X | X | X |  |  | X | X |
| - Victorian Institute Assessment – Gluteal (VISA-G)* | X |  |  | X | X | X |  |  | X | X |
| - Victorian Institute Assessment – Hamstring (VISA-H)* | X |  |  | X | X | X |  |  | X | X |
| - Victorian Institute Assessment – Patella (VISA-P)* | X |  |  | X | X | X |  |  | X | X |
| *Intervention Monitoring* |  |  |  |  |  |  |  |  |  |  |
| - Participant Monitoring Booklet (load, morning wellbeing, NPRS and sRPE) |  |  | X |  |  |  |  | X |  |  |
| *Pain* |  |  |  |  |  |  |  |  |  |  |
| - Brief Pain Inventory (BPI) | X |  |  | X | X | X |  |  | X | X |
| - McGill Pain Questionnaire – Short Form (MPQ) | X |  |  |  |  | X |  |  |  | X |
| - Pain Catastrophizing Scale (PCS) | X |  |  |  |  | X |  |  |  | X |
| *Psychosocial* |  |  |  |  |  |  |  |  |  |  |
| - Fatigue Assessment Scale (FAS) | X |  |  |  |  | X |  |  |  | X |
| - Tampa Scale of Kinesiophobia (TSK) | X |  |  | X | X | X |  |  | X | X |
| **Mechanistic Measures** |  |  |  |  |  |  |  |  |  |  |
| *Algometry* |  |  |  |  |  |  |  |  |  |  |
| - Pressure Pain Threshold | X | X | X | X | X | X | X | X | X | X |
| - Temporal Summation |  |  |  |  |  | X |  |  |  | X |
| *Blood Sampling* |  |  |  |  |  |  |  |  |  |  |
| - Beta-Endorphin |  |  |  |  |  | X |  | X^†^ | X | X |
| - Interluekin-6 |  |  |  |  |  | X | X |  | X | X |
| - Tumour Necrosis Factor-⍺ |  |  |  |  |  | X | X |  | X | X |
| **Physical Capacity Assessment** |  |  |  |  |  |  |  |  |  |  |
| - 5RM Lower Limb Strength | X |  |  |  | X | X |  |  |  | X |
| **Neuromuscular Performance** |  |  |  |  |  |  |  |  |  |  |
| - Isometric Mid-Thigh Pull | X |  |  |  | X | X |  |  | X | X |
| *Only the relevant injury-specific PROM will be completed with each study participant.  ^†^To be collected on Tuesday (x3) and Wednesday (x1) only.  Abbreviations: Brief Pain Inventory, BPI; Copenhagen Hip and Groin Outcome Score, HAGOS; Fatigue Assessment Scale, FAS; Foot and Ankle Disability Index, FADI; Health Anxiety Depression Scale, HADS; Knee Injury and Osteoarthritis Outcome Score, KOOS; Leeds Assessment of Neuropathic Symptoms and Signs, S-LANSS; Lower Extremity Function Scale, LEFS; McGill Pain Questionnaire – Short Form, MPQ; Musculoskeletal Health Questionnaire, MSK-HQ; Non-Arthritic Hips Score, NAHS; Numerical Pain Rating Scale, NPRS; Pain Catastrophizing Scale, PCS; Sessional Rate of Perceived Exertion, sRPE; Tampa Scale of Kinesiophobia, TSK; Victorian Institute Assessment – Achilles, VISA-A; Victorian Institute Assessment – Gluteal, VISA-G; Victorian Institute Assessment – Hamstring, VISA-H; Victorian Institute Assessment – Patella, VISA-P.  Phase One Timepoints – T0_P1_, Baseline (Day -3); T1 _P1_, Admission Day (Day 1); T2 _P1_, Daily (Days 1-5); T3 _P1_, End of Intervention (Day 5); T4 _P1_, Follow Up (Day 6).  Phase Two Timepoints – T0 _P2_, Baseline (Day -3); T1 _P2_, Admission Day (Day 1); T2 _P2_, Daily (Days 1-5, 6-10, 11-15); T3 _P2_, Start and End of Residential Rehabilitation Weeks (Days 1 & 5, 6 & 10, and 11 & 15); T4 _P2_, End of Intervention (Day 15). | | | | | | | | | | |
