# Supplementary material for: Efficacy and acceptability of different blood flow restriction training interventions during the rehabilitation of military personnel with lower limb musculoskeletal injuries: protocol for a two-phase randomised controlled trial
Source: BMJ Open. 2025 May 26;15(5):e096643. doi: 10.1136/bmjopen-2024-096643 (PMC12107567; doi:10.1136/bmjopen-2024-096643)
Supplement: online supplemental file 4 [file bmjopen-15-5-s004.docx]

| **Supplementary File 4.** Description of Patient Reported Outcome Measures | | |
| --- | --- | --- |
| *Timepoints* **^† ‡^** | *Outcome Measure or Instrument* | *Description* |
| †: T0, T3 & T4  ‡: T0, T3 & T4 | Copenhagen Hip and Groin Outcome Score (HAGOS)* (1) | The HAGOS is a patient administered, multi-dimensional, pain assessment tool commonly used to assess symptomatic and functional burden associated with hip and groin pathology (2). Scored from 0-100, lower scores on the HAGOS represent greater symptomatic and functional burden (3). The MCID per domain is 8, 8, 9, 10, 11, and 12 points for hip-related QOL, symptoms, pain, sport and recreation activities, ADL, and participation in physical activities, respectively (4). |
| ‡: T0 & T4 | Fatigue Assessment Scale (FAS) (5) | The FAS is a 10-question, self-reported questionnaire that covers mental and physical fatigue (6). The cumulative score for the FAS ranges from 10 to 50, with each question is scored from 1 (never) to 5 (always), with two requiring reverse scoring (7). A higher score indicates greater levels of fatigue, with scores >22 representing fatigue levels are greater than “healthy” or “normal” levels (6,8). The MCID for the FAS is 4-points (9). |
| †: T0, T3 & T4  ‡: T0, T3 & T4 | Foot and Ankle Disability Index (FADI)* (10) | The FADI is a 26-item ankle and foot specific, patient-reported, questionnaire which considers ADLs, function, pain and sleep (11). Each item is scored from 0 (unable to do/unbearable pain) to 4 (no difficulty at all/no pain), with a total point value reaching a maximum of 104; however, the PROM is scored as a percentage and a lower percentage indicates greater levels of disability and/or pain (11). To date, no MCID has been reported for the FADI outcome measure. |
| †: T0, T3 & T4  ‡: T0, T3 & T4 | Knee Injury and Osteoarthritis Outcome Score (KOOS)* (12) | The KOOS is a five domain, patient-administered questionnaire, used to monitor disease course and outcomes following knee injury/OA or surgery (13). The five domains, ADL, knee-related QOL, other symptoms, pain, and sport and recreation function, are scored on a 0-100 scale, with a higher score indicating better function and less symptoms (14). The MCID for the KOOS is 10-points for each domain (15). |
| †: T0, T3 & T4  ‡: T0, T3 & T4 | Leeds Assessment of Neuropathic Symptoms and Signs (S-LANSS)* (16) | The S-LANSS is a self-reported questionnaire with a primary aim of distinguishing pain of a neuropathic origin, without the need for clinical assessment (17). The questionnaire consists of 7 questions, with a higher score suggesting the pain is predominantly of neuropathic origin (18). To date, no MCID has been reported for the S-LANSS (19). |
| †: T0, T3 & T4  ‡: T0, T3 & T4 | Lower Extremity Function Scale (LEFS) (20) | The LEFS is a 20-item, patient-reported, questionnaire designed to measure the functional status of an individual with lower limb pathology (21). As each item is scored 0 (extreme difficulty/limitations) to 4 (no difficulty), a lower score is indicative of increased functional limitations relating to the lower limb pathology (22). The MCID for the LEFS was reported at 9 points (21). |
| †: T0, T3 & T4  ‡: T0, T3 & T4 | Non-Arthritic Hips Score (NAHS)* (23) | The NAHS is a 20-item, self-reported, questionnaire covering four domains (activities, function, pain and symptoms), in patients without arthritic pathology (24). The summative score for NAHS is between 0-100, with 100 representing a perfectly functioning hip (25). The MCID for this outcome measure is 8-points (26–28). |
| ‡: T0 & T4 | Tampa Scale of Kinesiophobia (TSK) (29) | The TSK is a 17-item questionnaire, developed in 1991, used to evaluate kinesiophobia in people with persistent musculoskeletal pain (30), by assessing activity avoidance (i.e., reflects beliefs of an activity that may result in an increase of pain, or cause injury) and somatic focus (i.e., reflects beliefs and underlying of serious conditions) (31,32). With each item scored 1 (strongly disagree) to 4 (strongly agree), a higher score denotes greater levels of fear of movement and/or re-injury (33). The MCID for TSK is a 4-point reduction (34). |
| †: T0, T3 & T4  ‡: T0, T3 & T4 | Victorian Institute Assessment – Achilles (VISA-A)* (35) | The VISA-A is a patient-reported questionnaire designed to assess the severity of Achilles tendinopathy (36). With questions focused on the domains of function in ADLs, pain, and sporting activity, the questionnaire is scored out of 100 (100 = asymptomatic); however, a score <60 is often seen in Achilles tendinopathy populations (37). The MCID is set at 14-points for mid-portion Achilles tendinopathy (38), and 6.5 points for insertional tendinopathy (39). |
| †: T0, T3 & T4  ‡: T0, T3 & T4 | Victorian Institute Assessment – Gluteal (VISA-G)* (40) | The VISA-G was designed to evaluate the severity of disability in greater trochanteric pain syndrome populations (41). Formulated in the same manner as other Victorian Institute of Sport Assessment tendinopathy measures, the VISA-G is scored out of 100, with a greater score associated with greater function and less symptom impairment (41). There is currently no MCID reported within the literature for the VISA-G (42). |
| †: T0, T3 & T4  ‡: T0, T3 & T4 | Victorian Institute Assessment – Hamstring (VISA-H)* (43) | The VISA-H was designed in the style of previous Victorian Institute of Sport Assessment tendinopathy outcome measures, but specifically to evaluate the severity of symptoms, function and ability to play sports in those with proximal hamstring tendinopathy (44). Scored out of 100, with higher scores being associated with greater function and less symptomatic impairments. A 4-point reduction was reported in the literature as being the threshold required to detect for true change when the standard error of measurement is considered (45); however, an MDIC of 22-points has been reported (44). |
| †: T0, T3 & T4  ‡: T0, T3 & T4 | Victorian Institute Assessment – Patella (VISA-P)* (46) | The VISA-P is an 8 question, patient-reported measure developed specifically to subjectively assess the severity of symptoms, function and ability to play sports in those with patella tendinopathy (47). Scored out of 100, a greater score is associated with greater function and less symptom impairment, with 0 being the theoretical minimum score (47). The MCID for the VISA-P is 13-points (48). |
| †: T0, T3 & T4  ‡: T0, T3 & T4 | Brief Pain Inventory (BPI) (49) | The BPI is a patient administered, multi-dimensional, pain assessment tool commonly used within musculoskeletal clinical practice (50,51). Higher scores on the 9-item short form indicate greater interference with function, or greater pain intensity (50). The MCID for the BPI is a 2-point reduction for average pain, pain interference and pain severity (52–54). |
| ‡: T0 & T4 | Pain Catastrophizing Scale (PCS) (55) | The PCS is a 13-item instrument that assesses helplessness, magnification and rumination to encompass the catastrophizing of musculoskeletal persistent pain (56). With each item scored 0 (not at all) to 4 (all the time), a higher score indicates greater catastrophizing of pain (57). A MCID of 8- and 11-points for no/low catastrophizers and catastrophizers (total score = >30 points), respectively, has been reported within the literature (58). |
| ‡: T0 & T4 | McGill Pain Questionnaire – Short Form (MPQ) (59) | The MPQ short form asks patients to rate 15 descriptors of affective and sensory feelings of pain (i.e., aching, sickening, throbbing) on a 1 (none) to 4 (severe) scale and completed a NPRS for average pain intensity (60). The affective and sensory section of the MPQ short form is graded from 15 to 45, with a higher score indicating a greater level of pain intensity and sensory variation; meanwhile, the NPRS of average pain is considered a separate entity (60). The MCID for this outcome measure is 5-points (61). |
| †: T2  ‡: T2 | Participant Monitoring Booklet | Daily Morning Wellbeing: Participants will complete a psychometric daily morning wellbeing questionnaire that covers 5 constructs (fatigue, general muscle soreness, mood, sleep quality, and stress levels) on a Likert scale from 1 (i.e., low mood, very fatigued) to 5 (i.e., very fresh, feeling great) that has previously been described within the literature (62). A summation of all 5 domains scores provides a total wellness score between 5-25.  Numerical Pain Rating Scale: Pain response and muscle discomfort will be assessed post-BFR exercise using Borg’s scale for pain (63), which ranged from 0 (nothing at all/no pain) to 10 (strongest intensity pain); however, patients were informed a score of 11 could be given if the pain was worse than any pain they had ever felt before, and is an approach commonly utilised within the literature relating to BFR exercise and perceptual response (64,65). The numerical pain rating scale (NPRS) has been shown to be sensitive to changes in pain and function in musculoskeletal (66) and persistent pain populations (67). An MCID of -1.5pts, -3pts, and -3.5pts is required for small, medium and large changes, respectively, for NPRS in musculoskeletal populations (68).  Rate of Perceived Exertion: Rate of perceived exertion response was assessed post-BFR exercise using the BORG CR10 scale, ranging from 0 (no exertion at all) to 10 (maximal exertion) (69). It was explained to participants that a rating of 0 meant they felt no exertion at all and a rating of 10 meant they were giving maximal effort and could not work any harder (69). |
| † Phase One: T0 = Pre-Admission; T1 = Admission; T2 = Daily (Pre-Intervention, Immediately Post-Intervention and 1-hour Post Intervention); T3 = End of Intervention Period; T4 = Follow Up.  ‡ Phase Two: T0 = Pre-Admission; T1 = Admission; T2 = Daily (Pre-Intervention, Immediately Post-Intervention and 1-hour Post Intervention); T3 = Start and End of Residential Weeks; T4 = End of Residential Week 3.  *Only the relevant injury-specific PROM will be completed with each study participant. | | |

**Associated References**

1. Giezen H, Stevens M, Van Den Akker-Scheek I, Reininga IHF. Validity and reliability of the Dutch version of the Copenhagen Hip And Groin Outcome Score (HAGOS-NL) in patients with hip pathology. PLoS One [Internet]. 2017 Oct 1 [cited 2023 Dec 7];12(10). Available from: https://pubmed.ncbi.nlm.nih.gov/29020040/

2. Thorborg K, Hölmich P, … RCBJS, 2011 undefined. The Copenhagen Hip and Groin Outcome Score (HAGOS): development and validation according to the COSMIN checklist. Br J Sports Med [Internet]. 2011 [cited 2023 Dec 1];45:478–91. Available from: https://www.researchgate.net/profile/Kristian-Thorborg/publication/51037519_The_Copenhagen_Hip_and_Groin_Outcome_Score_HAGOS_development_and_validation_according_to_the_COSMIN_checklist/links/00463520a7bb04edac000000/The-Copenhagen-Hip-and-Groin-Outcome-Score-HAGOS-development-and-validation-according-to-the-COSMIN-checklist.pdf

3. Thorborg K, Branci S, Stensbirk F, Jensen J, Hölmich P. Copenhagen hip and groin outcome score (HAGOS) in male soccer: reference values for hip and groin injury-free players. Br J Sports Med [Internet]. 2014 [cited 2024 Jun 4];48(7):557–9. Available from: https://pubmed.ncbi.nlm.nih.gov/23850734/

4. Winge S, Winge S, Kraemer O, Dippmann C, Hölmich P. Arthroscopic treatment for femoroacetabular impingement syndrome (FAIS) in adolescents-5-year follow-up. J Hip Preserv Surg [Internet]. 2021 [cited 2024 Jun 4];8:249–54. Available from: https://doi.org/10.1093/jhps/hnab051

5. Michielsen HJ, De Vries J, Van Heck GL. Psychometric qualities of a brief self-rated fatigue measure: The Fatigue Assessment Scale. J Psychosom Res [Internet]. 2003 Apr [cited 2023 Dec 7];54(4):345–52. Available from: https://pubmed.ncbi.nlm.nih.gov/12670612/

6. Hendriks C, Drent M, Elfferich M, De Vries J. The Fatigue Assessment Scale: Quality and availability in sarcoidosis and other diseases. Curr Opin Pulm Med. 2018;24(5):495–503.

7. Michielsen HJ, De Vries J, Van Heck GL. Psychometric qualities of a brief self-rated fatigue measure: The Fatigue Assessment Scale. J Psychosom Res. 2003 Apr 1;54(4):345–52.

8. De Vries J, Michielsen H, Van Heck GL, Drent M. Measuring fatigue in sarcoidosis: the Fatigue Assessment Scale (FAS). Br J Health Psychol [Internet]. 2004 Sep [cited 2024 Jul 4];9(Pt 3):279–91. Available from: https://pubmed.ncbi.nlm.nih.gov/15296678/

9. De Kleijn WPE, De Vries J, Wijnen PAHM, Drent M. Minimal (clinically) important differences for the Fatigue Assessment Scale in sarcoidosis. Respir Med [Internet]. 2011 Sep [cited 2024 Jul 4];105(9):1388–95. Available from: https://pubmed.ncbi.nlm.nih.gov/21700440/

10. Ibrahim T, Beiri A, Azzabi M, Best AJ, Taylor GJ, Menon DK. Reliability and validity of the subjective component of the American Orthopaedic Foot and Ankle Society clinical rating scales. J Foot Ankle Surg [Internet]. 2007 Mar [cited 2023 Dec 7];46(2):65–74. Available from: https://pubmed.ncbi.nlm.nih.gov/17331864/

11. Martin RL, Burdett RG, Irrgang JJ. Development of the foot and ankle disability index (FADI). Journal of Orthopaedic & Sports Physical Therapy. 1999;29(1).

12. Phatama KY, Aziz A, Bimadi MH, Oktafandi IGNAA, Cendikiawan F, Mustamsir E. Knee Injury and Osteoarthritis Outcome Score: Validity and Reliability of an Indonesian Version. Ochsner J [Internet]. 2021 [cited 2023 Dec 7];21(1):63. Available from: /pmc/articles/PMC7993429/

13. Roos EM, Roos HP, Lohmander LS, Ekdahl C, Beynnon BD. Knee Injury and Osteoarthritis Outcome Score (KOOS)—Development of a Self-Administered Outcome Measure. Journal of Orthopaedic & Sports Physical Therapy. 1998 Aug;28(2):88–96.

14. Collins NJ, Prinsen CAC, Christensen R, Bartels EM, Terwee CB, Roos EM. Knee Injury and Osteoarthritis Outcome Score (KOOS): systematic review and meta-analysis of measurement properties. Osteoarthritis Cartilage. 2016 Aug;24(8):1317–29.

15. Roos EM. 30 years with the Knee injury and Osteoarthritis Outcome Score (KOOS). Osteoarthritis Cartilage. 2024 Apr 1;32(4):421–9.

16. Koc R, Erdemoglu AK. Validity and reliability of the Turkish Self-administered Leeds Assessment of Neuropathic Symptoms and Signs (S-LANSS) questionnaire. Pain Med [Internet]. 2010 Jul [cited 2023 Dec 7];11(7):1107–14. Available from: https://pubmed.ncbi.nlm.nih.gov/20456071/

17. Bennett MI, Smith BH, Torrance N, Potter J. The S-LANSS score for identifying pain of predominantly neuropathic origin: Validation for use in clinical and postal research. J Pain. 2005 Mar;6(3):149–58.

18. Cho SI, Lee CH, Park GH, Park CW, Kim HO. Use of S-LANSS, a tool for screening neuropathic pain, for predicting postherpetic neuralgia in patients after acute herpes zoster events: A single-center, 12-month, prospective cohort study. Journal of Pain [Internet]. 2014 Feb 1 [cited 2024 Jun 6];15(2):149–56. Available from: http://www.jpain.org/article/S1526590013013011/fulltext

19. Fernández-de-Las-Peñas C, Arias-Buría JL, El Bachiri YR, Plaza-Manzano G, Cleland JA. Ultrasound-guided percutaneous electrical stimulation for a patient with cubital tunnel syndrome: a case report with a one-year follow-up. Physiother Theory Pract. 2022 Oct 3;38(10):1564–9.

20. Mehta SP, Fulton A, Quach C, Thistle M, Toledo C, Evans NA. Measurement Properties of the Lower Extremity Functional Scale: A Systematic Review. Journal of Orthopaedic and Sports Physical Therapy [Internet]. 2016 Mar 1 [cited 2023 Dec 7];46(3):200–16. Available from: https://www.jospt.org/doi/10.2519/jospt.2016.6165

21. Binkley JM, Stratford PW, Lott SA, Riddle DL. The Lower Extremity Functional Scale (LEFS): scale development, measurement properties, and clinical application. North American Orthopaedic Rehabilitation Research Network. Phys Ther. 1999 Apr;79(4):371–83.

22. Mehta SP, Fulton A, Quach C, Thistle M, Toledo C, Evans NA. Measurement Properties of the Lower Extremity Functional Scale: A Systematic Review. J Orthop Sports Phys Ther [Internet]. 2016 Mar 1 [cited 2024 Jun 6];46(3):200–16. Available from: https://pubmed.ncbi.nlm.nih.gov/26813750/

23. Dartus J, Putman S, Champagne G, Matache BA, Pelet S, Belzile EL. Validation of the French version of the Non-Arthritic Hip Score (NAHS) in 113 hip arthroscopy procedures. Orthopaedics & Traumatology: Surgery & Research. 2023 Nov 1;109(7):103683.

24. Dartus J, Putman S, Champagne G, Matache BA, Pelet S, Belzile EL. Validation of the French version of the Non-Arthritic Hip Score (NAHS) in 113 hip arthroscopy procedures. Orthopaedics & Traumatology: Surgery & Research. 2023 Nov 1;109(7):103683.

25. Ali MS, Khattak M, Metcalfe D, Perry DC. Radiological hip shape and patient-reported outcome measures in healed Perthes’ disease. Bone and Joint Journal. 2023 Jun 1;105-B(6):711–6.

26. Rosinsky PJ, Kyin C, Maldonado DR, Shapira J, Meghpara MB, Ankem HK, et al. Determining Clinically Meaningful Thresholds for the Nonarthritic Hip Score in Patients Undergoing Arthroscopy for Femoroacetabular Impingement Syndrome. Arthroscopy - Journal of Arthroscopic and Related Surgery [Internet]. 2021 Oct 1 [cited 2024 Jun 6];37(10):3113–21. Available from: http://www.arthroscopyjournal.org/article/S074980632100325X/fulltext

27. Meghpara MB, Yelton MJ, Glein RM, Malik MS, Rosinsky PJ, Shapira J, et al. Isolated Endoscopic Gluteus Medius Repair Can Achieve Successful Clinical Outcomes at Minimum 2-Year Follow-up. Arthrosc Sports Med Rehabil [Internet]. 2021 [cited 2024 Jun 6];3:e1697–704. Available from: https://doi.org/10.1016/j.asmr.2021.07.026

28. Bloom DA, Kaplan DJ, Kirby DJ, Buchalter DB, Lin CC, Fried JW, et al. The minimal clinically important difference for the nonarthritic hip score at 2-years following hip arthroscopy. Knee Surg Sports Traumatol Arthrosc [Internet]. 2022 Jul 1 [cited 2024 Jun 6];30(7):2419–23. Available from: https://pubmed.ncbi.nlm.nih.gov/34738159/

29. Dupuis F, Cherif A, Batcho C, Massé-Alarie H, Roy JS. The Tampa Scale of Kinesiophobia: A Systematic Review of Its Psychometric Properties in People With Musculoskeletal Pain. Clin J Pain [Internet]. 2023 May 13 [cited 2023 Dec 7];39(5):236–47. Available from: https://pubmed.ncbi.nlm.nih.gov/36917768/

30. Dupuis F, Cherif A, Batcho C, Massé-Alarie H, Roy JS. The Tampa Scale of Kinesiophobia: A Systematic Review of Its Psychometric Properties in People with Musculoskeletal Pain. Clinical Journal of Pain [Internet]. 2023 May 13 [cited 2024 Jun 17];39(5):236–47. Available from: https://journals.lww.com/clinicalpain/fulltext/2023/05000/the_tampa_scale_of_kinesiophobia__a_systematic.5.aspx

31. Vlaeyen JWS, Kole-Snijders AMJ, Boeren RGB, van Eek H. Fear of movement/(re)injury in chronic low back pain and its relation to behavioral performance. Pain [Internet]. 1995 [cited 2024 Jun 17];62(3):363–72. Available from: https://journals.lww.com/pain/fulltext/1995/09000/fear_of_movement__re_injury_in_chronic_low_back.13.aspx

32. Liu H, Huang L, Yang Z, Li H, Wang Z, Peng L. Fear of Movement/(Re)Injury: An Update to Descriptive Review of the Related Measures. Front Psychol [Internet]. 2021 Jul 7 [cited 2024 Jun 17];12:696762. Available from: https://pubmed.ncbi.nlm.nih.gov/

33. Miller RP, Kori SH, Todd DD. The Tampa Scale: A Measure of Kinesiophobia. Clin J Pain. 1991;7(1):51.

34. Rufa A, Beissner K, Dolphin M. The use of pain neuroscience education in older adults with chronic back and/or lower extremity pain. Physiother Theory Pract [Internet]. 2019 Jul 3 [cited 2024 Jun 17];35(7):603–13. Available from: https://www.tandfonline.com/doi/abs/10.1080/09593985.2018.1456586

35. Robinson JM, Cook JL, Purdam C, Visentini PJ, Ross J, Maffulli N, et al. The VISA-A questionnaire: a valid and reliable index of the clinical severity of Achilles tendinopathy. Br J Sports Med [Internet]. 2001 Oct 1 [cited 2023 Dec 7];35(5):335–41. Available from: https://bjsm.bmj.com/content/35/5/335

36. Robinson JM, Cook JL, Purdam C, Visentini PJ, Ross J, Maffulli N, et al. The VISA-A questionnaire: a valid and reliable index of the clinical severity of Achilles tendinopathy. Br J Sports Med [Internet]. 2001 [cited 2024 Jun 19];35(5):335. Available from: /pmc/articles/PMC1724384/

37. Iversen JV, Bartels EM, Langberg H. THE VICTORIAN INSTITUTE OF SPORTS ASSESSMENT – ACHILLES QUESTIONNAIRE (VISA-A) – A RELIABLE TOOL FOR MEASURING ACHILLES TENDINOPATHY. Int J Sports Phys Ther [Internet]. 2012 Feb [cited 2024 Jun 19];7(1):76. Available from: /pmc/articles/PMC3273883/

38. Lagas IF, van der Vlist AC, van Oosterom RF, van Veldhoven PLJ, Reijman M, Verhaar JAN, et al. Victorian Institute of Sport Assessment-Achilles (VISA-A) Questionnaire—Minimal Clinically Important Difference for Active People With Midportion Achilles Tendinopathy: A Prospective Cohort Study. Journal of Orthopaedic & Sports Physical Therapy. 2021 Oct;51(10):510–6.

39. McCormack J, Underwood F, Slaven E, Cappaert T. THE MINIMUM CLINICALLY IMPORTANT DIFFERENCE ON THE VISA‐A AND LEFS FOR PATIENTS WITH INSERTIONAL ACHILLES TENDINOPATHY. Int J Sports Phys Ther [Internet]. 2015 Oct [cited 2024 Jun 19];10(5):639. Available from: /pmc/articles/PMC4595917/

40. Jorgensen JE, Fearon AM, Mølgaard CM, Kristinsson J, Andreasen J. Translation, validation and test-retest reliability of the VISA-G patient-reported outcome tool into Danish (VISA-G.DK). PeerJ [Internet]. 2020 [cited 2023 Dec 7];2020(3). Available from: /pmc/articles/PMC7060749/

41. Fearon AM, Ganderton C, Scarvell JM, Smith PN, Neeman T, Nash C, et al. Development and validation of a VISA tendinopathy questionnaire for greater trochanteric pain syndrome, the VISA-G. Man Ther. 2015 Dec 1;20(6):805–13.

42. Clifford C, Paul L, Syme G, Millar NL. Isometric versus isotonic exercise for greater trochanteric pain syndrome: a randomised controlled pilot study What are the new findings? BMJ Open Sp Ex Med [Internet]. 2019 [cited 2024 Jun 19];5:558. Available from: http://dx.doi.org/10.1136/bmjsem-2019-000558

43. Cacchio A, De Paulis F, Maffulli N. Development and validation of a new visa questionnaire (VISA-H) for patients with proximal hamstring tendinopathy. Br J Sports Med [Internet]. 2014 [cited 2023 Dec 7];48(6):448–52. Available from: https://pubmed.ncbi.nlm.nih.gov/23470447/

44. Cacchio A, De Paulis F, Maffulli N. Development and validation of a new visa questionnaire (VISA-H) for patients with proximal hamstring tendinopathy. Br J Sports Med. 2014 Mar;48(6):448–52.

45. Korakakis V, Whiteley R, Kotsifaki A, Stefanakis M, Sotiralis Y, Thorborg K. A systematic review evaluating the clinimetric properties of the Victorian Institute of Sport Assessment (VISA) questionnaires for lower limb tendinopathy shows moderate to high-quality evidence for sufficient reliability, validity and responsiveness—part II. Knee Surgery, Sports Traumatology, Arthroscopy [Internet]. 2021 Sep 1 [cited 2024 Jun 19];29(9):2765–88. Available from: https://link.springer.com/article/10.1007/s00167-021-06557-0

46. Zwerver J, Kramer T, Van Den Akker-Scheek I. Validity and reliability of the Dutch translation of the VISA-P questionnaire for patellar tendinopathy. BMC Musculoskelet Disord [Internet]. 2009 Aug 11 [cited 2023 Dec 7];10(1):1–5. Available from: https://bmcmusculoskeletdisord.biomedcentral.com/articles/10.1186/1471-2474-10-102

47. Visentini PJ, Khan KM, Cook JL, Kiss ZS, Harcourt PR, Wark JD. The VISA score: an index of severity of symptoms in patients with jumper’s knee (patellar tendinosis). Victorian Institute of Sport Tendon Study Group. J Sci Med Sport [Internet]. 1998 [cited 2024 Jun 19];1(1):22–8. Available from: https://pubmed.ncbi.nlm.nih.gov/9732118/

48. Hernandez-Sanchez S, Hidalgo MD, Gomez A. Responsiveness of the VISA-P scale for patellar tendinopathy in athletes. Br J Sports Med [Internet]. 2014 [cited 2024 Jun 19];48(6):453–7. Available from: https://pubmed.ncbi.nlm.nih.gov/23012320/

49. Mendoza T, Mayne T, Rublee D, Cleeland C. Reliability and validity of a modified Brief Pain Inventory short form in patients with osteoarthritis. European Journal of Pain [Internet]. 2006 May 1 [cited 2023 Dec 7];10(4):353–353. Available from: https://onlinelibrary.wiley.com/doi/full/10.1016/j.ejpain.2005.06.002

50. Stanhope J. Brief Pain Inventory review. Occup Med (Chic Ill) [Internet]. 2016 Aug 1 [cited 2024 Jun 3];66(6):496–7. Available from: https://dx.doi.org/10.1093/occmed/kqw041

51. Jumbo SU, MacDermid JC, Kalu ME, Packham TL, Athwal GS, Faber KJ. Measurement Properties of the Brief Pain Inventory-Short Form (BPI-SF) and the Revised Short McGill Pain Questionnaire-Version-2 (SF-MPQ-2) in Pain-related Musculoskeletal Conditions: A Systematic Review Protocol. Archives of Bone and Joint Surgery [Internet]. 2020 Mar 1 [cited 2024 Jun 3];8(2):131. Available from: /pmc/articles/PMC7191979/

52. Mathias SD, Crosby RD, Qian Y, Jiang Q, Dansey R, Chung K. Estimating minimally important differences for the worst pain rating of the brief pain inventory-short form. Journal of Supportive Oncology. 2011 Mar;9(2):72–8.

53. Mease PJ, Spaeth M, Clauw DJ, Arnold LM, Bradley LA, Russell IJ, et al. Estimation of minimum clinically important difference for pain in fibromyalgia. Arthritis Care Res (Hoboken). 2011 Jun;63(6):821–6.

54. Song CY, Chen CH, Chen TW, Chiang HY, Hsieh CL. Assessment of Low Back Pain: Reliability and Minimal Detectable Change of the Brief Pain Inventory. American Journal of Occupational Therapy [Internet]. 2022 May 1 [cited 2023 Dec 1];76(3). Available from: /ajot/article/76/3/7603205040/23260/Assessment-of-Low-Back-Pain-Reliability-and

55. Franchignoni F, Giordano A, Ferriero G, Monticone M. Measurement precision of the Pain Catastrophizing Scale and its short forms in chronic low back pain. Sci Rep [Internet]. 2022 Dec 1 [cited 2023 Dec 7];12(1):12042. Available from: /pmc/articles/PMC9283330/

56. Franchignoni F, Giordano A, Ferriero G, Monticone M. Measurement precision of the Pain Catastrophizing Scale and its short forms in chronic low back pain. Scientific Reports 2022 12:1 [Internet]. 2022 Jul 14 [cited 2024 Jun 6];12(1):1–9. Available from: https://www.nature.com/articles/s41598-022-15522-x

57. Sullivan MJL, Bishop SR, Pivik J. The Pain Catastrophizing Scale: Development and Validation. Psychol Assess. 1995;7(4):524–32.

58. Monticone M, Portoghese I, Rocca B, Giordano A, Campagna M, Franchignoni F. Responsiveness and minimal important change of the Pain Catastrophizing Scale in people with chronic low back pain undergoing multidisciplinary rehabilitation. Eur J Phys Rehabil Med [Internet]. 2022 Feb 1 [cited 2023 Dec 1];58(1):68. Available from: /pmc/articles/PMC9980597/

59. Adelmanesh F, Jalali A, Attarian H, Farahani B, Ketabchi SM, Arvantaj A, et al. Reliability, Validity, and Sensitivity Measures of Expanded and Revised Version of the Short-Form McGill Pain Questionnaire (SF-MPQ-2) in Iranian Patients with Neuropathic and Non-Neuropathic Pain. Pain Medicine (United States) [Internet]. 2012 Dec 1 [cited 2023 Dec 7];13(12):1631–6. Available from: https://dx.doi.org/10.1111/j.1526-4637.2012.01517.x

60. Melzack R. The short-form McGill pain questionnaire. Pain. 1987 Aug 1;30(2):191–7.

61. Strand LI, Ljunggren AE, Bogen B, Ask T, Johnsen TB. The Short-Form McGill Pain Questionnaire as an outcome measure: test-retest reliability and responsiveness to change. Eur J Pain [Internet]. 2008 Oct [cited 2024 Jul 4];12(7):917–25. Available from: https://pubmed.ncbi.nlm.nih.gov/18289893/

62. Hooper SL, Mackinnon LT. Monitoring Overtraining in Athletes: Recommendations. Sports Medicine [Internet]. 1995 Oct 7 [cited 2024 Jul 23];20(5):321–7. Available from: https://link.springer.com/article/10.2165/00007256-199520050-00003

63. Heath EM. Borg’s Perceived Exertion and Pain Scales. Medicine& Science in Sports & Exercise. 1998 Sep;30(9):1461.

64. Jessee MB, Dankel SJ, Buckner SL, Mouser JG, Mattocks KT, Loenneke JP. The Cardiovascular and Perceptual Response to Very Low Load Blood Flow Restricted Exercise. Int J Sports Med [Internet]. 2017 Jul 1 [cited 2023 Dec 8];38(8):597–603. Available from: http://www.thieme-connect.de/products/ejournals/html/10.1055/s-0043-109555

65. Dankel SJ, Buckner SL, Counts BR, Jessee MB, Mouser JG, Mattocks KT, et al. The acute muscular response to two distinct blood flow restriction protocols. Physiol Int [Internet]. 2017 Mar 1 [cited 2024 May 28];104(1):64–76. Available from: https://pubmed.ncbi.nlm.nih.gov/28361570/

66. Hefford C, Lodge S, Elliott K, Abbott JH. Measuring patient-specific outcomes in musculoskeletal clinical practice: a pilot study. New Zealand Journal of Physiotherapy [Internet]. 2008 Jul 1 [cited 2024 May 28];36(2):41–9. Available from: https://go.gale.com/ps/i.do?p=AONE&sw=w&issn=03037193&v=2.1&it=r&id=GALE%7CA181366666&sid=googleScholar&linkaccess=fulltext

67. Cheatham SW, Kolber MJ, Mokha M, Hanney WJ. Concurrent validity of pain scales in individuals with myofascial pain and fibromyalgia. J Bodyw Mov Ther. 2018 Apr 1;22(2):355–60.

68. Abbott JH, Schmitt J. Minimum Important Differences for the Patient-Specific Functional Scale, 4 Region-Specific Outcome Measures, and the Numeric Pain Rating Scale. Journal of Orthopaedic & Sports Physical Therapy. 2014 Aug;44(8):560–4.

69. Williams N. The Borg Rating of Perceived Exertion (RPE) scale. Occup Med (Chic Ill) [Internet]. 2017 Jul 1 [cited 2024 May 25];67(5):404–5. Available from: https://dx.doi.org/10.1093/occmed/kqx063
