# Supplementary material for: Efficacy and acceptability of different blood flow restriction training interventions during the rehabilitation of military personnel with lower limb musculoskeletal injuries: protocol for a two-phase randomised controlled trial
Source: BMJ Open. 2025 May 26;15(5):e096643. doi: 10.1136/bmjopen-2024-096643 (PMC12107567; doi:10.1136/bmjopen-2024-096643)
Supplement: Supplementary file 5 [file bmjopen-2024-096643.reviewer_comments.pdf]

PEER REVIEW HISTORY

BMJ Open publishes all reviews undertaken for accepted manuscripts. Reviewers are asked to complete a checklist review form and are provided with free text boxes to elaborate on their assessment. These free text comments are reproduced below.

ARTICLE DETAILS

Title (Provisional)

The efficacy and acceptability of different blood flow restriction training interventions during the rehabilitation of military personnel with lower limb musculoskeletal injuries: protocol for a two-phase randomised controlled trial.

Authors

Gray, Luke; Coppack, Russ J; Barker-Davies, Robert; Cassidy, Robyn P; Bennett, Alexander; Caplan, Nick; Atkinson, Gavin; Bradshaw, Lauren; Chauhan, Janisha; Lunt, Kieran M; Hughes, Luke; Ladlow, Peter

---

VERSION 1 - REVIEW

**Reviewer**	**1**
**Name**	**Dubé, Marc-Olivier**
**Affiliation**	**Université Laval**
**Date**	**01-Dec-2024**
**COI**	**None**

Thanks for the opportunity to review this RCT protocol about the use of different blood flow restriction training interventions for the management of lower limb MSK disorders in a military population. I commend the authors for their detailed protocol.

Please find my comments below.

My main issues are:

- Ensuring that T0, T1, T2, T3, T4 for both phases are clear for the reader

- The limited sample size and short-term follow-up are not discussed enough and this should be reflected in statements made in discussion.

- Phase 2 should be referred to as a pilot trial. See my comments about sample size calculation.

Title/abstract

- I appreciate the authors wanting to provide as much information as possible in the title. I wonder if “primarily limited by pain” could be removed. Given that the primary reason of consultation for MSK injuries is pain, it kind of goes without saying.

- I believe post-operative should be hyphenated

- Please add clear primary purpose of trial in background section of abstract.

- Page 2, Line 28: comparing is repeating.

- Please add projected sample sizes in abstract

- Page 2, Line 31: The 2 timepoints for baseline (T0/T1) and completion of intervention (T3/T4) when presented without additional details.

Strengths/limitations

- I suggest removing second bullet point and replacing by one stating the limitations with such a “short-term” follow-up.

Introduction

- Exhaustive literature review. The main weakness of the introduction is the lack of a justification as to why a short-term pain reduction is clinically relevant. Most of the evidence highlights within-session reduction of pain.

Study aims

- Can the authors provide hypothesis for both aims.

Methods

- Can the authors provide rationale for choosing 40% and 80% ?

- Can the authors specify if physiotherapist and exercise

- rehabilitation instructor involved are the same for all patients? Can they provide more information about them? (ie. years of experience).

- I appreciate the description the authors have provided in Table. To enable replication, could the authors complete the TIDIEr template/checklist and provide it as a supplementary file please?

- Please provide more details about stratification by pathology? What are the categories?

- Page 11, line 12-13: The clinicians providing intervention will be unblinded (no choice), but those collecting outcomes data such as mechanistic outcomes, strength and neuromuscular performance could be blinded to treatment allocation. I suggest that the authors try to aim for this. I don’t see why those collecting data should be unblinded.

- Please provide rationale for 10% dropout assumption.

- Stratification can help with increasing power but mostly when stratification factors have a high pronostic value. Is this the case? Please provide evidence for this. Otherwise, I would be careful with statement.

- Can the authors provide more details about the data used to calculate effect size in sample size calculation. How did they end up with 0.64. This effect size magnitude appears large, is there evidence from other trials that you should expect this. With a lower expected effect size, your trial would require a bigger sample in order to be adequately powered. Even when considering the repeated measurements, the sample size of 11/arm seems low.

- Please justify the 80% threshold for compliance. Is there evidence of a minimally adequate dose.

- I appreciate the authors wanting to conduct sub-group analyses (e.g., sex) but with such a limited sample size, I wonder how this will be feasible. Those analyses will probably end up being grossly underpowered and thus not relevant.

- Page 12, line 29: Post intervention adverse events between groups using Fisher's exact test. Missing words

- Please ensure description of timepoints is clearer. What does T2, T3, T4 represents. A bit confusing at times with different ones used for both phases.

Discussion

- Line 18: "multi-system beneficial adaptions being increasingly reported." Can the authors provide any example?

- Line 33-34: Strong statement given limited sample size and short-term follow-up. Suggest removing or being more nuanced.

- Consider adding a section in discussion about any methodological limitations and ways that they will be addressed.

Tables/figures

- Table 2: Acronyms need to be defined below table

- Table 3: T4 is not the same for both phases but having two T4 makes it confusing. Can authors use another way of defining timepoints. Same comment applies for rest of text.

- Figure 1: Define BFR40 and BFR80.

- Figure 1: Provide more details about T1, T3 and T4 timepoints.

- Figure 2: Define BFR40 and BFR80.

- Figure 3: Define BFR and CON

- Figure 3: Provide more details about T1 and T4 timepoints.

Reviewer 2

Name **Maselli, Filippo**

Affiliation **University of Rome La Sapienza, Department Human Neurosciences**

Date **18-Mar-2025**

COI **None**

I realize that authors have many journals to consider when they want to publish their work, so I appreciate your interest in BMJ Open; I am really happy to be able to write in a positive way. It is evident that you have put a great deal of effort into this project and I want to praise your efforts. Fortunately, the actual contribution from your study protocol for a two-phase randomised controlled trial is strong, a well-constructed and the manuscript as currently written suggests that it might be suitable for sharing information about this topic like as efficacy and acceptability of different blood flow restriction training. The protocol that you reported, needs minor edits. I would like to thank you for give me an opportunity to consider this work for publication.

It may be that the you would like to consider resubmitting it, in which case I hope that the comments from my review may help you to revise it before resubmitting it. These comments are given below.

- I suggest adding what the authors mean by persistent pain, what definition they refer to and inserting the citation

- I suggest adding or explaining better the characteristics of the BFR, i.e. device used, etc.

VERSION 1 - AUTHOR RESPONSE

Reviewer: 1

My main issues are:

- Ensuring that T0, T1, T2, T3, T4 for both phases are clear for the reader

We appreciate that this may be confusing for readers, so we have added specific details and days to the table and figure legends to allow easier interpretation. An example includes:

- *Phase One Timepoints – $T0_{P1}$, Baseline (Day -3); $T1_{P1}$, Admission Day (Day 1); $T2_{P1}$, Daily (Days 1-5); $T3_{P1}$, End of Intervention (Day 5); $T4_{P1}$, Follow Up (Day 6).*

- *Phase Two Timepoints – $T0_{P2}$, Baseline (Day -3); $T1_{P2}$, Admission Day (Day 1); $T2_{P2}$, Daily (Days 1-5, 6-10, 11-15); $T3_{P2}$, Start and End of Residential Rehabilitation Weeks (Days 1 & 5, 6 & 10, and 11 & 15); $T4_{P2}$, End of Intervention (Day 15).*

- The limited sample size and short-term follow-up are not discussed enough and this should be reflected in statements made in discussion.

We thank the reviewer for this comment. The reference for the effect size was not added and this has now been amended (P11). Additionally, in your main comments it is mentioned about “Phase Two” being called a pilot study. We agree that the sample size is small and should be considered a pilot study, but for continuity we coined the studies “Phase One” and Phase Two”. We take guidance from Julious (2005, DOI:10.1002/pst.185) who states that a

sample size of N=12 per arm is justified and adequate for pilot studies (which we meet with our actual sample size calculation).

- Phase 2 should be referred to as a pilot trial. See my comments about sample size calculation.
We thank the reviewer for this comment. We agree that the sample size is small and should be considered a pilot study, but for continuity we coined the studies "Phase One" and Phase Two". We take guidance from Julious (2005, DOI:10.1002/pst.185) who states that a sample size of N=12 per arm is justified and adequate for pilot studies (which we meet with our actual sample size calculation).

Title/abstract
- I appreciate the authors wanting to provide as much information as possible in the title. I wonder if "primarily limited by pain" could be removed. Given that the primary reason of consultation for MSK injuries is pain, it kind of goes without saying.
Amended.

- I believe post-operative should be hyphenated
Amended.

- Please add clear primary purpose of trial in background section of abstract.
We thank the reviewer for this comment. Added P1, Ln 22-25.

- Page 2, Line 28: comparing is repeating.
Removed.

- Please add projected sample sizes in abstract
Added P1, Ln30/32.

- Page 2, Line 31: The 2 timepoints for baseline (T0/T1) and completion of intervention (T3/T4) when presented without additional details.
We thank the reviewer for this comment. T0-4 have been removed to avoid confusion within the abstract. This should be made clearer later within the study (e.g., T4 is end of intervention/follow up in Phase One/Two, respectively).

Strengths/limitations
- I suggest removing second bullet point and replacing by one stating the limitations with such a "short-term" follow-up.
We thank the reviewer for this comment. The strength and limitations have been completely altered as per Editors comments. This, however, has been noted on P2, Ln15-24.

Introduction
- Exhaustive literature review. The main weakness of the introduction is the lack of a justification as to why a short-term pain reduction is clinically relevant. Most of the evidence highlights within-session reduction of pain.
We understand the reviewers comments and agree that the justification presented was not strong enough within the literature review. This has now been adjusted (P5, Ln34-37 and P6, Ln1).

Study aims
- Can the authors provide hypothesis for both aims.
Added on P6, Ln24-26/33-34.

Methods

- Can the authors provide rationale for choosing 40% and 80% ?

Both, 40 and 80%, pressures have been utilised within previous hypoalgesia literature, and were discussed, specifically, in the literature. This has now been referred to in the methodology section (P13, Ln3-4).

- Can the authors specify if physiotherapist and exercise rehabilitation instructor involved are the same for all patients? Can they provide more information about them? (ie. years of experience).

Due to the nature of the military, physiotherapists and ERIs will rotate on 2 year cycles so there is no guarantee that they are the same for all participants. However, all physiotherapists and ERI's receive the same training (per profession) to ensure continuity and best practice across UK Defence Rehabilitation. This has been reflected on P7.

- I appreciate the description the authors have provided in Table. To enable replication, could the authors complete the TIDIEr template/checklist and provide it as a supplementary file please?

We are unsure which table is being referred to, however a TIDIEr template has been completed and added as a supplementary file.

- Please provide more details about stratification by pathology? What are the categories?

We thank the reviewer for this comment. Pathology was added in error, this should've stated age and has now been amended on P10, Ln4.

- Page 11, line 12-13: The clinicians providing intervention will be unblinded (no choice), but those collecting outcomes data such as mechanistic outcomes, strength and neuromuscular performance could be blinded to treatment allocation. I suggest that the authors try to aim for this. I don't see why those collecting data should be unblinded.

Due to this protocol being part of a PhD programme embedded within a small team, the individual delivering the intervention is also collecting all outcome measures on their own and is the only individual within the department to be trained in the collection of all outcome measures.

- Please provide rationale for 10% dropout assumption.

We thank the reviewer for this comment. The 10% drop out assumption was utilised on the back of a previous BFR RCT (Hughes et al., 2019; DOI: 10.1007/s40279-019-01137-2), and inferred from the recruitment of other Defence Medical Rehabilitation Centre RCTs (Cassidy et al., 2023; DOI: 10.1186/s12891-023-06693-3 // Coppack et al., 2016; DOI: 10.1186/s12891-016-1309-z // Barker-Davies et al., 2017; DOI: 10.1186/s12891-017-1564-7).

- Stratification can help with increasing power but mostly when stratification factors have a high pronostic value. Is this the case? Please provide evidence for this. Otherwise, I would be careful with statement.

Removed.

- Can the authors provide more details about the data used to calculate effect size in sample size calculation. How did they end up with 0.64. This effect size magnitude appears large, is there evidence from other trials that you should expect this. With a lower expected effect size, your trial would require a bigger sample in order to be adequately powered. Even when considering the repeated measurements, the sample size of 11/arm seems low.

We thank the reviewer for this comment. Apologies, the reference for the effect size was not added and this has now been amended (P11). Additionally, in your main comments it is mentioned about “Phase Two” being called a pilot study. We agree that the sample size is small and should be considered a pilot study, but for continuity we coined the studies “Phase One” and Phase Two”. We take guidance from Julious (2005, DOI:10.1002/pst.185) who states that a sample size of N=12 per arm is justified and adequate for pilot studies (which we meet with our actual sample size calculation).

- Please justify the 80% threshold for compliance. Is there evidence of a minimally adequate dose.
We thank the reviewer for this comment. This was standard practice for other ADMR RCTs so carried forward.

- I appreciate the authors wanting to conduct sub-group analyses (e.g., sex) but with such a limited sample size, I wonder how this will be feasible. Those analyses will probably end up being grossly underpowered and thus not relevant.
We thank the reviewer for this comment. We understand and have now removed.

- Page 12, line 29: Post intervention adverse events between groups using Fisher’s exact test. Missing words
We thank the reviewer for this comment, this has now been amended.

- Please ensure description of timepoints is clearer. What does T2, T3, T4 represents. A bit confusing at times with different ones used for both phases.
We thank the reviewer for this comment. We appreciate that this may be confusing for readers, so we have added specific details and days to the tables and figure legends to allow easier interpretation.

Discussion
- Line 18: “multi-system beneficial adaptions being increasingly reported.” Can the authors provide any example?
We thank the reviewer for this comment. Examples added on P20, Ln25-26.

- Line 33-34: Strong statement given limited sample size and short-term follow-up. Suggest removing or being more nuanced.
Removed.

- Consider adding a section in discussion about any methodological limitations and ways that they will be addressed.
We thank the reviewer for this comment. This section has now been added highlighting limitations in study design (P21, Ln7-19).

Tables/figures
- Table 2: Acronyms need to be defined below table
Abbreviations removed.

- Table 3: T4 is not the same for both phases but having two T4 makes it confusing. Can authors use another way of defining timepoints. Same comment applies for rest of text.
We thank the reviewer for this comment. We appreciate that this may be confusing for readers, so we have added specific details and days to the table legend to allow easier interpretation.

- Figure 1: Define BFR40 and BFR80.

This has now been defined within the Figure legend (P31, Ln17-21).

- Figure 1: Provide more details about T1, T3 and T4 timepoints.
A full description has now been added to the figure legend (P31, Ln17-21).

- Figure 2: Define BFR40 and BFR80.
This has now been defined within the Figure legend (P31, Ln22-29).

- Figure 3: Define BFR and CON
This has now been defined within the Figure legend (P31, Ln 30-34).

- Figure 3: Provide more details about T1 and T4 timepoints.
A full description has now been added to the figure legend (P31, Ln 30-34)

Reviewer: 2
- I suggest adding what the authors mean by persistent pain, what definition they refer to and inserting the citation
Persistent pain is already defined, with citation, in paragraph two of the background (P3, Ln15).

- I suggest adding or explaining better the characteristics of the BFR, i.e. device used, etc.
We thank the reviewer for this comment. The device used was already described within the "experimental intervention" section of the methodology; however, we have added more specific details (e.g., cuff characteristics) (P13, Ln7-17).

VERSION 2 - REVIEW

**Reviewer**	**1**
**Name**	**Dubé, Marc-Olivier**
**Affiliation**	**Université Laval**
**Date**	**16-Apr-2025**
**COI**	

The authors have addressed all of my previous comments.

I still believe that whenever they talk about their phase 2 RCT, they should identify it as a pilot RCT. This applies to title and o

VERSION 2 - AUTHOR RESPONSE

Response to Reviewer 1
Thank you for all of your support reviewing our manuscript and confirming that we have addressed all of your previous comments.

I have shared your view/opinion regarding the use of the word 'pilot' with the authorship team. The unanimous viewpoint is that we do not change the title or refer to the second RCT as a pilot for the reasons provided within the previous round of review... "We take guidance from Julious (2005, DOI:10.1002/pst.185) who states that a sample size of N=12 per arm is justified and adequate for pilot studies (which we meet with our actual sample size calculation)."
